# Bryophytes and the symbiotic microorganisms, the pioneers of vegetation restoration in karst rocky desertification areas in southwestern China

**DOI:** 10.1007/s00253-019-10235-0

**Published:** 2019-12-10

**Authors:** Wei Cao, Yuanxin Xiong, Degang Zhao, Hongying Tan, Jiaojiao Qu

**Affiliations:** 1 College of Life Sciences, Guizhou University, Guiyang, 550025 China; 2 The Key Laboratory of Plant Resources Conservation and Germplasm Innovation in Mountainous Region (Ministry of Education), Institute of Agro-Bioengineering and College of Life Sciences, Guizhou University, Guiyang, 550025 China; 3 Guiyang A’ha Lake National Wetland Park Management Division, Guiyang, 550002 China; 4 College of Tea Sciences, Guizhou University, Guiyang, 550025 China

**Keywords:** Bryophyte diversity, Drought resistance, Bacterial and fungal communities, High-throughput sequencing

## Abstract

In karst rocky desertification areas, bryophytes coexist with algae, bacteria, and fungi on exposed calcareous rocks to form a bryophyte crust, which plays an irreplaceable role in the restoration of karst degraded ecosystems. We investigated the biodiversity of crust bryophytes in karst rocky desertification areas from Guizhou Province, China. A total of 145 species in 22 families and 56 genera were identified. According to frequency and coverage, seven candidate dominant mosses were screened out, and five drought-resistant indexes of them were measured. *Hypnum leptothallum*, *Racopilum cuspidigerum*, and *Hyophila involuta* have high drought adaptability. We explored the interactions between two dominant mosses (*H. leptothallum*, *H. involuta*) and the structure of microbial communities in three karst rocky desertification types. Microbial diversity and function analysis showed that both moss species and karst rocky desertification types affect microbial communities. Moss species much more strongly affected the diversity and changed the community composition of these microbial groups. Bacteria were more sensitive in the microbiome as their communities changed strongly between mosses and drought resistance factors. Moreover, several species of fungi and bacteria could be significantly associated with three drought-resistant indexes: Pro (free proline content), SOD (superoxide dismutase activity), and POD (peroxidase activity), which were closely related to the drought adaptability of mosses. Our results enforced the potential role of moss-associated microbes that are important components involved in the related biological processes when bryophytes adapted to arid habitats, or as one kind of promoters in the distribution pattern of early mosses succession in karst rocky desertification areas.

**Electronic supplementary material:**

The online version of this article (10.1007/s00253-019-10235-0) contains supplementary material, which is available to authorized users.

## Introduction

Karst rocky desertification is a process of land degradation involving serious soil erosion, extensive exposure of basement rocks, a drastic decrease in soil productivity, and the appearance of a desert-like landscape (Wang et al. 2004a). As a result of intensive land use, this kind of rocky landscape is caused by degrading the fragile subtropical karst environment (Wang et al. 2004b). According to the karst rock exposure rate and vegetation coverage, rocky desertification is divided into five types of desertification: potential, slightly, moderately, severely, and very severely (Xiong et al. 2002). The karst rocky desertification has tremendously affected hydrologic, soil, and ecologic conditions at various scales and consequently causes various geologic hazards, such as droughts, floods, landslides, and land subsidence. In addition, profoundly, the carbon balance and regional climate conditions affected by the expansion of karst rocky desertification threaten the living conditions of people in the area (Jiang et al. 2014). In Southwest China, Guizhou Province is a central area to various provinces, including Yunnan, Guangdong, Chongqing, Hunan, Hubei, and Sichuan, and the Guangxi Zhuang autonomous region, and is the largest ecologically fragile karst region in the world (Yuan 2014; Jiang et al. 2014; Xiong and Chi 2015; Fang and Li 2017; Chen et al. 2019). Karst rocky desertification seriously hinders the sustainable development of local economy, society, and ecology.

Promoting ecological restoration and helping farmers out of poverty in Karst rocky desertification areas are important goals that local governments have been striving to achieve over the past decade in China (Zhang et al. 2015a, b). Currently, in Southwest China, the ways to control karst rocky desertification mainly include (1) returning farmland to forests and grasslands, (2) intensive management and development of ecological agriculture and animal husbandry, (3) integrated developmental model of agriculture and forestry, (4) planting economic trees, and (5) ecological migration and so on (Xiao et al. 2014; Fang and Li 2017; Cheng et al. 2019). With continuous resources and financial support from the central and local governments in China as well as the practices of some control and restore measures, the overall karst rocky desertification area in Southwest China has been reduced by 7.4% from 2005 to 2011 (0.12 million km^2^) (Jiang et al. 2014; Fang and Li 2017). Even so, by the end of 2016, the area of karst rocky desertification in China’s karst areas was ca. 0.10 million km^2^ (http://www.mnr.gov.cn/dt/ywbb/201812/t20181217_2379630.html); thus, challenges obviously remain in combating karst rocky desertification (Zhang et al. 2016). Therefore, it is very urgent to seek more sustainable measures to control karst rocky desertification and restore the ecological environment.

Bryophytes, the most primitive higher plants and the pioneer taxa in the positive succession of natural ecosystems, are one of only a few desiccation-tolerant plants that thrive on calcareous rocks and can grow on karst rocky desertification areas (Wu 1998; Yuan 2008). Most of them are poikilohydric plants, and one of the most remarkable features of bryophytes is their ability to survive in dry environments (Kallio and Karenlampi 1975; Proctor et al. 2007). Some desiccation-tolerant taxa have unique physical structures and strong adaptive mechanisms, which make them capable of growing in extremely harsh environments (e.g., high temperature, seasonal drought, and calcium-rich) (Chen 1963; Giordano et al. 1993; Hamerlynck et al. 2000; Oliver et al. 2000; Gao et al. 2018). In karst rocky desertification areas, bryophytes usually coexist with algae, bacteria, fungi, and rhizosphere fine particles on exposed rocks (with high temperatures, seasonal droughts, and are rich in calcium) to form bryophyte crust. Bryophyte crusts not only have unique ecological functions but also have high biomass and obvious ecological effects, which play an irreplaceable role in the restoration and reconstruction of degraded ecosystems (Belnap and Lange 2013; Pointing 2016; Cheng et al. 2019). In karst rocky desertification areas, their ecological functions include (1) accelerating the disintegration of broken rocks; (2) increasing water retention and fertility of rock surfaces by absorbing water, dust particles, and nutrients from the atmosphere; (3) promoting mineral decomposition and soil formation by releasing CO_2_, organic acid, and carbonic anhydrase; (4) balancing subsoil acidity and alkalinity and improving its physical and chemical properties, subsequently creating conditions for invasion and settlement of other species; and (5) affecting the composition of microbial communities and their growth and reproduction (Cao and Yuan 1999; Guo and Cao 2001; Li et al. 2005; Maik 2005; Li et al. 2007; Zheng et al. 2009; Xiao et al. 2014). However, in the karst rocky desertification areas of Southwest China, the biodiversity survey of bryophytes within a larger scale is very limited. There are several studies on the community of bryophytes in single areas (such as Goujiang karst bauxite and Huajiang karst plateau in Guizhou Province, Guogai, in the mountain area of Yunnan Province), and the connection between some moss species and water retention, erosion resistance, soil enzyme activity, and soil formation has been preliminarily studied (Huang and Xie 2003; Li and Zhang 2009; Liu et al. 2009; Chen and Zhang 2010; Wang et al. 2011; Li et al. 2013, 2015; Zhang et al. 2013, 2014; Zhang et al. 2015a, b, 2016; Yin et al. 2016; Cong et al. 2017; Liu et al. 2018). However, the selection of optimal species, symbiosis between bryophytes and microorganisms, artificial culture techniques, etc. have not attracted enough attention. Studies focusing on a single rocky desertification type or sampling point cannot capture bryophyte fluctuations that profoundly change bryophyte species composition throughout an area. Therefore, the survey of bryophyte diversity in different rocky desertification regions is a necessary prerequisite for the further application of dominant bryophyte species.

Microbial fungi and bacteria are ubiquitous, occurring in highly enriched concentrations in bryophytes, and investigation of bacterial communities has attracted global attention in recent years (Garcia-Pichel et al. 2001; Yeager et al. 2004; Abed et al. 2010; Steven et al. 2014; Blay et al. 2017; Maier et al. 2018). Nevertheless, exploration of the diversity and community of non-lichenized fungi with bryophytes is still quite limited (Bates et al. 2012; Zhang et al. 2016; Liu et al. 2017). Most of these studies focused on the diversity of moss-related bacterial communities in deserts, forests, grasslands, and alpine regions, while the structure and function of bryophyte-symbiotic microbial communities in karst rocky desertification habitats have not been reported (Opelt et al. 2007; Tang et al. 2016; Ma et al. 2017). Studies have shown that *Proteobacteria* and *Actinobacteria* were the two most abundant phyla associated with mosses under different habitats, and large amounts of unclassified bacteria could interact with the moss. In addition, the structure and function of the symbiotic fungal community with bryophytes are often neglected. We suspect that the abundant moss-associated microbes might also be important components involved in related biological processes, such as adaptation to aquatic, anaerobic, and even extreme drought environments. To better understand the entire microbiome responses to different classes of karst rocky desertification and to disentangle the role of key microbial taxa in microbiome communities of dominant species of bryophytes, an integrative study of all microbial groups is needed.

Guizhou Province is the central area of the largest ecologically fragile karst region in the world. Here, we investigated the biodiversity of crust bryophytes in different karst rocky desertification regions in Guizhou Province, where a total of 1620 species of taxa have been recorded (Xiong 2014a, b; Xiong and Cao 2018). Then the drought adaptation mechanism of 7 candidate dominant bryophytes was explored. Finally, we performed a holistic analysis of 2 moss microbiome members including bacteria and fungi using group-specific high-throughput sequencing approaches on samples obtained from three major representative karst rocky desertification classes. The results of this study not only enhanced our understanding of the interactions between mosses and their symbiotic microorganisms and the factors that influence microbial communities in mosses but also will be helpful for better use and development of the dominant moss-bryophytic crust technique in environmental restoration for karst rocky desertification.

## Materials and methods

### Field quadrat setup and samplings

According to aerial photos, satellite photo observation, and ground investigation in different periods, the field quadrats were located across a latitudinal gradient in Guizhou Province, China, from southwest to northeast. The sites were chosen to represent the three main classes of karst rocky desertification areas in Guizhou Province and consisted of 14 sample sites, including very severely areas (Ve, such as Xingyi and Anshun City), severely areas (Se, such as Panzhou City and Yanhe County), and slightly areas (Sl, such as Guiyang City and Bijie City). The main classes of karst rocky desertification areas in Guizhou Province and the geographic location of all sample quadrats are shown in Supplemental Table S1 and Supplemental Fig. S1.

The sample quadrat, which was dependent on the actual situation, was randomly set, and each quadrat was 1 m × 1 m (for the stone face that was not square, the square projection of the spatial horizontal plane was taken as the sampling range) and all kinds of moss samples were collected in sterile centrifuge tubes for further processing.

To evaluate the composition of moss-associated bacterial and fungal communities, two dominant mosses, *Hypnum leptothallum* (Müll. Hal.) Paris (Supplemental Fig. S2) and *Hyophila involuta* (Hook.) A. Jaeger (Supplemental Fig. S3) (denoted as Hyp and Hyo, respectively), including plants of them and underlying rhizosphere fine particles were collected from three different sites in Guihzou Province, China (Ve-Zerong Village, Xingyi City; Se-Zhongjie Village, Yanhe County; Sl-Qiaotao Village, Guiyang City; shown in Supplemental Fig. S1, Supplemental Table S1 and S2), on Oct. 25–27, 2018. Approximately 10 g of each species was sampled. Three samples of the same species were collected from each sample quadrat and mixed into one composite sample. Three composite samples of each species were collected from each site. In total, 18 composite samples were collected and immediately carried back to the laboratory and stored at 4 °C until further processing.

### Survey of dominant bryophytes

To investigate the biodiversity of bryophytes in karst rocky desertification areas in Guizhou Province, field specimens were collected for species identification and statistical analysis. The statistical indicators of the dominant species included (1) the species frequency, that is, the number of quadrats of a species existence in/the total quadrats × 100; (2) the ratio of *S*_N_/*S*, where *S*_N_ indicated the number of quadrats with the species N collected in this survey and *S* was denoted as the total number of samples; *S* in this study was 675; (3) the ratio of *S*_N_/*N*_a_, which was used as the sub-standard, where *N*_a_ was denoted as the number of sample sites that the species N was collected from; and (4) *N*_a_/*T*_a_, which is the assessment index, where *T*_a_ was denoted as the total number of sample sites; *T*_a_ was 14 in this study. The above 4 indexes were used to analyze the dominant species by statistics, and finally, the dominant species of natural distribution in karst rocky desertification areas of Guizhou Province were screened out. All collected specimens were kept in the natural museum herbarium of Guizhou University (GACP).

### Physiological properties of dominant bryophytes

To investigate the correlation between the physiological properties and ecological adaptation ability of the bryophyte dominant species in karst rocky desertification areas, we selected 7 mosses with high frequency and coverage in all field quadrats to measure the drought resistance index, including *Meteorium polytrichum* Dozy & Molk., *H. involuta* (Hook.) A. Jaeger, *Thuidium kanedae* Sakurai, *Racopilum cuspidigerum* (Schwägr.) Ångström, *Anomodon viticulosus* (Hedw.) Hook. & Taylor, *H. leptothallum* (Müll. Hal.) Paris, *Plagiomnium cuspidatum* (Hedw.) T. J. Kop. All tested mosses were placed in clean Petri dishes and cultured in an incubator at a temperature of 20 ± 2 °C and a natural light cycle. After 35 days, they were harvested for the determination of physiological indicators of drought resistance.

#### Membrane permeability measurement

The determination method was based on Shi et al. (2006) and slightly improved. Briefly, since the leaves of moss are very small, the volume scale method was used to ensure that the volume of the sampled leaves was consistent. Moss leaves with a volume equivalent of 0.5 mL were placed in a 50-mL glass beaker, rinsed with distilled water 3 times. Then, the beakers were filled with 30 mL of distilled water and allowed to stand in the dark for 24 h at room temperature. The electrical conductivity (EC1) of the bathing solution was determined at the end of the incubation period. After that, the beakers were heated in a water bath at 95 °C for 20 min and then cooled to room temperature, and the electrical conductivity (EC2) was measured. Electrolyte leakage was calculated as the percentage of EC1/EC2.

#### Determination of free proline content

Free proline (Pro) was extracted with sulfosalicylic acid from 1 g of fresh moss leaf, and the proline concentration was colorimetrically quantified according to the ninhydrin colorimetric method (Bates et al. 1973).

#### Determination of malondialdehyde content

The thiobarbituric acid (TBA) colorimetric method was used to determine the malondialdehyde (MDA) of moss. Refer to Zhang and Fan (2007) for the detailed experimental methods.

#### Superoxide dismutase activity and peroxidase activity test

The superoxide dismutase (SOD) activity of moss was determined by the nitroblue tetrazolium (NBT) photochemical reduction method, and the guaiacol method was used to determine the peroxidase (POD) activity. The protocol used was in reference to Chen and Zhang (2016).

#### Comprehensive evaluation method of drought resistance index

The subordinate function value method was used to evaluate the drought resistance of different mosses (Li et al. 2006). The average value of the above drought resistance membership function of all the indexes of each moss was calculated. The larger the mean value of the membership function, the stronger the drought resistance was. In addition, Data Processing System software (DPS, v15.10) (http://www.dpsw.cn/index.html) was used to directly calculate the correlation order of each index.

### Microbial diversity analysis

#### Sample collection

In this study, 18 samples of *H. leptothallum* and *H. involuta* (denoted as Hyp and Hyo, respectively) (including the plants and the rhizosphere fine particles of them) were obtained through field sampling and stored at 4 °C. Then, these samples (mixed with plants and rhizosphere fine particles) were used for the subsequent analysis.

#### DNA extraction, PCR assays, and high-throughput sequencing

Microbial DNA was extracted from the two moss samples using the E.Z.N.A.® Soil DNA Kit (Omega Bio-Tek, Norcross, GA, USA) according to the manufacturer’s protocols. The final DNA concentration and purification were determined by a NanoDrop 2000 UV-vis spectrophotometer (Thermo Scientific, Wilmington, USA), and DNA quality was checked by 1% agarose gel electrophoresis. The V3-V4 hypervariable regions of bacteria 16S rRNA and the internal transcribed spacer (ITS) regions were amplified with primers 338F (5′-ACTCCTACGGGAGGCAGCAG-3′) and 806R (5′-GGACTACHVGGGTWTCTAAT-3′), ITS1F (CTTGGTCATTTAGAGGAAGTAA) and ITS2R (GCTGCGTTCTTCATCGATGC), respectively, by a thermocycler PCR system (GeneAmp 9700, Carlsbad, ABI, USA). The PCRs were conducted using the following program: 3 min of denaturation at 95 °C, 27 cycles of 30 s at 95 °C, 30 s for annealing at 55 °C, and 45 s for elongation at 72 °C, and a final extension at 72 °C for 10 min. PCR reactions were performed in triplicate in 20 μL mixtures containing 4 μL of 5 × FastPfu Buffer, 2 μL of 2.5 mM dNTPs, 0.8 μL of each primer (5 μM), 0.4 μL of FastPfu Polymerase, and 10 ng of template DNA. The resulting PCR products were extracted from a 2% agarose gel, further purified using the AxyPrep DNA Gel Extraction Kit (Axygen Biosciences, Union City, CA, USA) and quantified using QuantiFluor™-ST (Promega, Madison, USA) according to the manufacturer’s protocol. Purified amplicons were pooled in equimolar amounts and paired-end sequenced (2 × 300) on an Illumina MiSeq platform (Illumina, San Diego, USA) according to the standard protocols by Majorbio Bio-Pharm Technology Co. Ltd. (Shanghai, China). The raw reads were deposited into the NCBI Sequence Read Archive (SRA) database (SRA accession: PRJNA557311; the accession link: https://www.ncbi.nlm.nih.gov/sra/PRJNA557311).

#### Processing of sequencing data

Raw fastq files were quality-filtered by Trimmomatic (Aachen, Germany) and merged by FLASH (San Francisco, USA) with the following criteria: (i) The reads were truncated at any site receiving an average quality score < 20 over a 50-bp sliding window. (ii) Sequences whose overlap was longer than 10 bp were merged according to their overlap with mismatch no more than 2 bp. (iii) Sequences of each sample were separated according to barcodes (exactly matching) and primers (allowing 2 nucleotide mismatching), and reads containing ambiguous bases were removed. Operational taxonomic units (OTUs) were clustered with a 97% similarity cut-off using UPARSE (version 7.1 http://drive5.com/uparse/) with a novel “greedy” algorithm that performed chimera filtering and OTU clustering simultaneously. The taxonomy of each 16S rRNA and ITS gene sequence was analyzed by the RDP Classifier algorithm (http://rdp.cme.msu.edu/) against Silva (SSU123) 16S rRNA and Unite (Release 7.0 http://unite.ut.ee/index.php), respectively, using a confidence threshold of 70%.

### Bioinformatics analysis

The alpha diversity of bacterial and fungal communities was calculated based on Faith’s phylogenetic metric at the OTU level. Nonmetric multidimensional scaling (NMDS) was used to visualize the dissimilarity of beta diversity based on the unweighted Unifrac distance across different moss species on three rocky desertification types (Quast et al. 2013). Microbial ecological functions were analyzed using the FAPROTAX (http://www.loucalab.com/archive/FAPROTAX/lib/php/index.php?section=Home) database, which annotated bacterial functions, and the FUNGuild (http://www.funguild.org/) database was used to predict the ecological function of fungal communities (the confidence levels were probable and highly probable, respectively) (Louca et al. 2016; Nguyen et al. 2016). Shared and unique OTUs among two moss species at three rocky desertification types were used to generate a Venn diagram. Bray-Curtis dissimilarity values were calculated to reveal the relationships among all samples based on the bacterial and fungal communities (Xue et al. 2017). Redundancy analysis (RDA) or canonical correspondence analysis (CCA) were performed with vegan in R to determine which environmental variables best explained the taxon distribution across communities (Zhao et al. 2016). The co-occurrence of genera in microbial communities across two moss species was analyzed using NetworkX in the Python package of the R platform (3.5.1) (https://pypi.org/project/networkx/). To reduce network complexity and facilitate the identification of the core moss community, we selected the top 50 bacterial and fungal genera of two moss species from all karst rocky desertification types for further analysis. To explore the pairwise associations, correlation scores (Spearman correlation) were calculated, and only significant correlations (*P* < 0.05) were retained for the downstream procedure (Faust and Raes 2012). The topology property parameters of the network, the degree, betweenness centrality, and closeness centrality of each node in the network were calculated by the plug-in Network Analyzer in Cytoscape (Assenov et al. 2007).

### Statistics analysis

Statistical analyses were performed in Origin Pro 8.0 (OriginLab, Northampton, MA, USA). One-way ANOVA was used to analyze differences in physiological and biochemical properties of seven mosses and alpha diversity among two mosses species and karst rocky desertification types. Statistical differences were considered significant at *P* < 0.05. Permutation multivariate analysis of variance (PERMANOVA) was employed to assess the significance of the influential factors that differentiated the moss drought resistance indexes and microbiomes (packages: vegan, adonis function) (Zhao et al. 2019). Linear discriminant analysis effect size (LEfSe) was performed to investigate potential biomarkers (across five taxonomic levels, from phylum to genus for bacterial and fungal communities) within microbiomes specifically enriched in one of the moss species and rocky desertification type based on *P* < 0.05 and an LDA score > 2.0 (Segata et al. 2011).

## Results

### The dominant species of moss in different karst rocky desertification habitats

In this study, a total of 185 field quadrats were investigated, 675 samples were collected, and a total of 145 moss species (including 1 subspecies and 2 varieties) in 22 families and 56 genera were identified. A detailed inventory of all collected moss specimens was made (Supplemental Table S1 and S3). A total of 22 families of mosses were identified, including the dominant families *Pottiaceae*, *Bryaceae*, *Hypnaceae*, and *Brachytheciaceae*, which all contain more than 10 species. There are 11 species of liverworts in 3 families (Supplemental Table S3). The proportion analysis shows that most of the collected moss specimens are mainly distributed in the temperate zone, with a few tropical distribution species.

In Table 1, the ratio of the top 20 dominant species in the natural distribution of karst rocky desertification samples was screened based on the early screening standard, and the frequency was 0.621, indicating that these species are the main components of the moss groups in karst rocky desertification land. We have considered firstly the frequency (including *S*_N_/*S*, *S*_N_/*N*_a_, and *N*_a_/*T*_a_), and 9 species, namely *H. leptothallum*, *Didymodon fallax*, *H. involuta*, *T. kanedae*, *Hyophila javanica*, *Bryum argenteum*, *Trichostomum crispulum*, *Palamocladium euchloron*, and *Didymodon constrictus* var. *constrictus*, have been screened out. However, among them, *H. leptothallum*, *H. involuta*, and *T. kanedae* have greater coverage. Beyond that, among excluded species, *A. viticulosus*, *M. polytrichum*, *P. cuspidatum*, and *R. cuspidigerum* have also greater coverage. When we think about determining the dominant species, we should consider comprehensively the frequency and coverage in natural environment. So, we have selected the 7 species as candidate dominant species to explore the adaptation mechanism of bryophytes in karst rocky desertification habitats, namely *M. polytrichum*, *H. involuta*, *T. kanedae*, *R. cuspidigerum*, *A. viticulosus*, *H. leptothallum*, and *P. cuspidatum*.

**Table 1:** Top 20 dominant bryophytes collected from karst rocky desertification areas

No.	Species	Species frequency	*S*_N_/*S*	*S*_N_/*N*_a_	*N*_a_/*T*_a_
1	*Hypnum leptothallum*	39.5	0.118	6.636	0.786
2	*Didymodon fallax*	21.6	0.059	3.333	0.857
3	*Bryum recurvulum*	14.6	0.040	3.222	0.357
4	*Hyophila involuta*	13.5	0.037	5.400	0.857
5	*Thuidium kanedae*	13.5	0.037	3.571	0.500
6	*Hyophila javanica*	12.4	0.034	2.000	0.643
7	*Bryum argenteum*	11.9	0.033	2.556	0.714
8	*Trichostomum crispulum*	10.8	0.030	2.200	0.643
9	*Palamocladium euchloron*	10.8	0.030	2.500	0.571
10	*Didymodon constrictus* var. *constrictus*	9.7	0.027	2.143	0.643
11	*Barbula unguiculata*	8.1	0.022	1.875	0.500
12	*Anomodon viticulosus*	8.1	0.022	5.000	0.214
13	*Ptychomitrium gardneri*	8.1	0.022	1.875	0.571
14	*Meteorium polytrichum*	8.1	0.022	1.857	0.571
15	*Plagiomnium cuspidatum*	6.5	0.018	3.000	0.286
16	*Bryum funkii*	5.9	0.016	1.833	0.429
17	*Weissia breviseta*	5.4	0.015	1.667	0.429
18	*Racopilum cuspidigerum*	5.4	0.015	2.500	0.286
19	*Bryum algovicum*	4.3	0.012	1.333	0.429
20	*Eurhynchium eustegium*	4.3	0.012	2.000	0.286
Total	–	–	0.621	49.865	10.572

### Moss physiological properties of drought resistance

To explore the adaptation mechanism of bryophytes in karst rocky desertification habitats, through five drought resistance index analyses, we found that the drought resistance ability of 7 candidate dominant mosses in karst rocky desertification habitats had different results when evaluating different indexes (Supplemental Table S2; Supplemental Fig. S4). This is related to the different ways in which different species adapt to the arid environment (Li et al. 2002). For example, the membrane permeability and malondialdehyde content mainly reflect the ability of plants to resist changes in membrane structure. The enhancement of Pro content, SOD activity, and POD activity mainly reflects the adaptability of plants to adversity by inhibiting catabolism (Souza et al. 2016). Therefore, a comprehensive analysis of these multiple indicators is needed to truly reflect the drought resistance of the seven mosses.

Using the fuzzy membership function method, five indexes that are closely related to drought resistance were selected to comprehensively evaluate the drought resistance of seven mosses. From Table 2, the order of drought resistance of the seven mosses was *H. leptothallum* > *R. cuspidigerum* > *H. involuta* > *A. viticulosus* > *P. cuspidatum* > *T. kanedae* > *M. polytrichum*. This sequence was consistent with the ratio of the number of samples to the number of plots, which indirectly reflects the biomass of the natural distribution. In addition, using DPS software, the gray relation analysis was carried out with the average membership function value in the comprehensive evaluation results as the generating sequence (Table 3). The results indicated that the five physiological indicators of resistance selected in this study had the most important impact on the drought resistance of the seven mosses adapted to karst rocky desertification habitats. The free Pro content, POD activity, and SOD activity were the most important, followed by plasma membrane permeability, and MDA content had the least impact.

**Table 2:** Resist-drought comprehensive evaluation form of seven mosses

Items	Species						
	*H. leptothallum*	*R. cuspidigerum*	*H. involuta*	*A. viticulosus*	*P. cuspidatum*	*T. kanedae*	*M. polytrichum*
Z(MP)	0.8346	0.5984	0.3226	0.8035	1.0000	0.5258	0.0000
Z(Pro)	1.0000	0.469	0.0201	0.2791	0.2464	0.8827	0.0000
Z(MDA)	1.0000	0.9877	0.5825	0.9767	0.3315	0.0000	0.2864
Z(SOD)	0.9618	1.0000	0.8835	0.0000	0.2927	0.3726	0.6273
Z(POD)	0.3549	0.5374	1.0000	0.3351	0.1929	0.0109	0.0000
Average $\overline{\mathrm{Zij}}$	0.8303	0.7185	0.5617	0.4789	0.4127	0.3584	0.1827
Order	1	2	3	4	5	6	7

**Table 3:** The correlation between 5 resistance indexes

Index	Free Pro	POD activity	SOD activity	Membrane permeability	MAD
Correlation coefficient	0.4951	0.4863	0.4495	0.2523	0.2039
Order	1	2	3	4	5

Through the comprehensive analysis, *H. leptothallum*, *R. cuspidigerum*, and *H. involuta* were classified as high drought-resistant species, and *P. cuspidatum* and *M. polytrichum* were low drought-resistant species. These results showed that the 7 mosses could reduce the water to adapt to drought stress mainly through the accumulation of free proline increasing the solute concentration inside the cell. At the same time, by increasing the activity of various antioxidant enzymes in cells, the destruction of the cell membrane of these mosses species by reactive oxygen species was eliminated, which could protect the integrity of the cell membrane structure against drought stress. These mosses have evolved drought resistance mechanisms from morphological and physiological aspects in the long-term adaptation in karst rocky desertification habitats.

### Moss microbiomes

#### Bacterial community

**Fig. 1:**
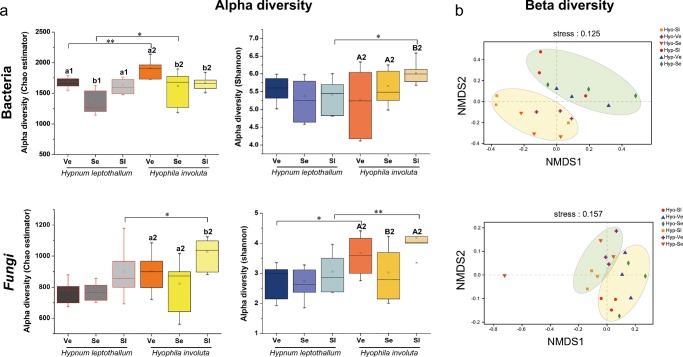
Alpha and beta diversity of the moss bacterial and fungal community. Chao and Shannon indexes were calculated based on phylogenetic distance at the OTU level and displayed in boxplots. The difference in alpha diversity among two moss species in different karst rocky desertification habitats was tested by one-way ANOVA (*P* < 0.05), and the labeled asterisk implies the significant difference (*P* < 0.05) of alpha diversity observed in the two species at the same areas. Beta diversity was analyzed by nonmetric multidimensional scaling (NMDS) based on unweighted UniFrac phylogenetic distance metrics at the OTU level and displayed in a scatter diagram

**Fig. 2:**
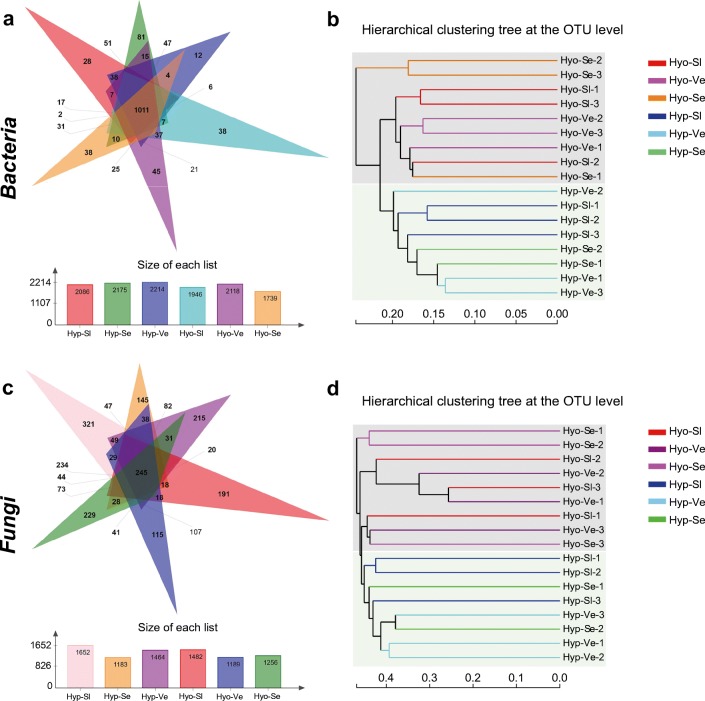
The composition similarities and differences of microorganisms in moss samples at different karst rocky desertification habitats. **a**, **c** Venn diagram of exclusive and shared bacterial and fungal OTUs (at the 3% evolutionary distance). **b**, **d** Hierarchical cluster analysis using pairwise unweighted UniFrac distances. Hyp1, Hyp2, and Hyp3 are respectively represented *H. leptothallum* in severely area, very severely area, and slightly area; Hyo1, Hyo2, and Hyo3 are respectively represented *H. involuta* in severely area, very severely area, and slightly area

To investigate the structure and function of the symbiotic microbial community, two moss species with better drought resistance in different karst rocky desertification habitats were analyzed by means of macro genome sequencing. After splicing and filtering the sequencing data, the remaining 1,021,116 sequences were clustered into 2892 bacterial OTUs obtained from 18 moss samples. Two species exhibited significant (*P* < 0.01 and *P* < 0.05) changes in the alpha diversity (Chao index) of the bacterial community in the Ve and Se areas, respectively (Fig. 1a). The results of bacterial alpha diversity show that *H. involuta* has a higher bacterial diversity than *H. leptothallum*. Furthermore, there was no overall difference (*P* > 0.05) in the Shannon index of the bacterial community between Ve and Se, except for the Sl area (*P* < 0.05) (Fig. 1a). Unconstrained ordination of the bacterial community matrix (Bray-Curtis distance-based NMDS) (Fig. 2b) demonstrated that the samples were divided according to moss species, and this grouping appeared to be significant according to PERMANOVA analyses (*P* = 0.001) (Table 4). Notably, grouping according to rocky desertification classes was not significant (*P* > 0.05).

**Table 4:** The effects of drought resistance index, karst rocky desertification type and moss species on the differentiation of bacterial and fungal communities based on PERMANOVA

		SOD	Pro	POD	MP	MAD	Karst rocky desertification type	Moss species	Karst rocky desertification type × moss species
Bacterial community	*R*^2^	0.164	0.163	0.106	0.097	0.072	0.198	0.263	0.427
	*P*	0.001	0.002	0.02	0.058	0.221	0.006	0.001	0.001
Fungal community	*R*^2^	0.108	0.109	0.086	0.068	0.085	0.122	0.204	0.314
	*P*	0.017	0.02	0.081	0.287	0.081	0.017	0.004	0.268

Taxonomic analysis revealed that *Actinobacteria* and *Proteobacteria* were most abundant across all samples at the phylum level, with relatively stable proportions of 19.54–45.83% and 19.21–31.20%, respectively, which are similar to some previous studies on bryophytes collected from different areas (Ma et al. 2017; Tang et al. 2016). Additionally, *Cyanobacteria*, *Chloroflexi*, *Acidobacteria*, *Bacteroidetes*, and *Verrucomicrobia* were the dominant phyla from the whole areas of two moss species according to relative abundance (> 1% was identified as a dominant bacterium) (Supplemental Fig. S5a and S6a). At the class level, *Actinobacteria*, *Cyanobacteria*, *Alphaproteobacteria*, and *Acidobacteria* dominated (Supplemental Fig. S6b). A total of 534 genera were obtained at the genus level from the bacterial community. The top 50 classified genera and 18 samples were both hierarchically clustered based on the Bray-Curtis similarity index (Supplemental Fig. S6c). It can be observed that samples within the same species were relevant. The cluster analysis showed that the top 50 genera, such as *Pseudonocardia*, *Cyanobacteria*, *Micromonospora*, and *Sphingomonas*, were the most representative bacterial genera in two moss species.

In addition, a Venn diagram demonstrated that OTUs differed among the two moss species at different rocky desertification types (Fig. 2a). The number of site-specific OTUs ranged from 12 (Hyp-Ve) to 81 (Hyp-Se). In addition, a total of 1011 OTUs were shared among all samples; these were defined as the core microorganisms of the bacterial community. Hierarchical cluster analysis revealed that the 18 bacterial community samples clustered into two groups that corresponded very well to the two moss species (Fig. 2b). Clustering indicated that the structure of the bacterial community was closely related to bryophyte species rather than habitat. Therefore, the results suggest that moss species may be a major factor in changing microbial community differentiation in karst rocky desertification ecosystems.

**Fig. 3:**
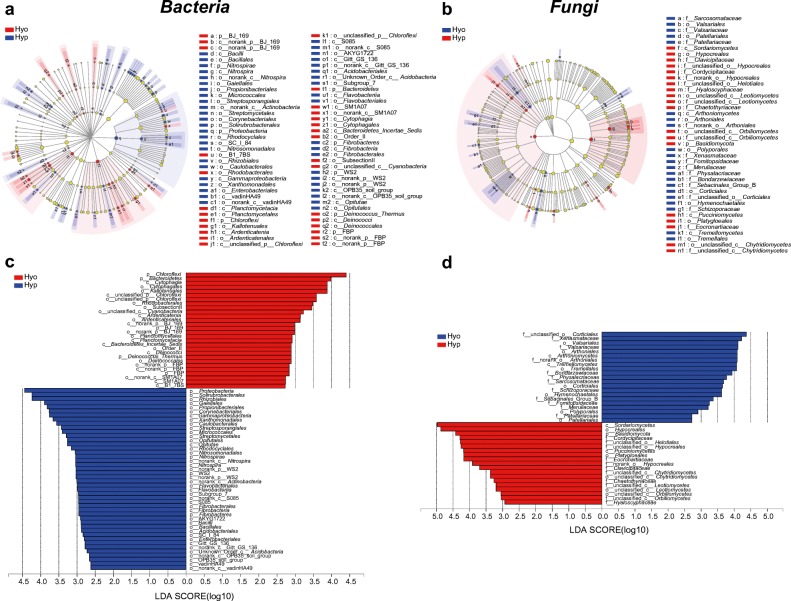
LEfSe results revealed bacteria (**a**, **c** from phylum level to order level) and fungi biomarkers (**b**, **d** from phylum level to family level) that were sensitive to Hyp and Hyo in the three karst rocky desertification areas. There are five circular rings in the cladogram, each circular ring deposit all taxa within a taxonomic level; the circular ring from inside to outside represents phylum, class, and order (or with an additional family), respectively. The node on the circular ring represents a taxon affiliating within the taxonomic level. Taxa that had significantly higher relative abundance in a certain moss species were color-coded within the cladogram according to the Bacterial and fungal Ribosomal Reference (PR2) taxonomy

The LEfSe analysis revealed that 54 biomarkers affiliating with 6 phyla were sensitive to two moss species (*P* < 0.05, LDA > 2.0; Fig. 3a; Supplemental Table S4). These biomarkers accounted for 6.99% of all taxa retrieved. For instance, two taxa within the class *Alphaproteobacteria* were more sensitive to Hyp, while the genus *Craurococcus* within this order was significantly enriched in Hyo. Two taxa within the order *Rhizobiales* and three genera within the class *Actinobacteria* were most susceptible to Hyp species, and the genus *Pseudonocardia* and three unclassified genera within the order *Kallotenuales* were enriched in the Hyo. In addition, the bacteria (from phylum level to order level) that sensitive to three karst rocky desertification areas (slightly, severely, and very severely) of two moss species were analyzed (Supplemental Fig. S7). The sensitive biomarkers of bacteria were significantly different at above two influencing factors.

#### Fungal community

After splicing and filtering the sequencing data, the remaining 1,179,721 sequences were clustered into 3351 fungal OTUs. Good’s coverage index of all samples reached over 99.61%, indicating that the sequencing depth met the requirements of reflecting all information of moss species. The alpha diversity of the microbial community in moss is shown in Supplemental Table S5. The alpha diversity (Chao and Shannon indexes) of the two species showed significant (*P* > 0.05) changes in the fungal community in the Sl areas (Fig. 1a). Moreover, the beta diversity of fungal communities was also strongly affected by the moss species (Fig. 1b).

The fungal community associated with the two moss species showed high taxonomic diversity. Altogether, the fungi were classified into 7 phyla, 27 classes, and 550 genera. At the phylum level, the dominant phyla were *Ascomycota* and candidate divisions, and the fungi remained unassigned at the family level in *Pleosporales* and *Capnodiales* (Supplemental Fig. S5b, S8a, and S8b). However, the results showed that the dominant species were the same in different samples at the phylum level, but the relative abundance was different. Interestingly, the entomopathogenic fungi *Cordycipitaceae* and *Ophiocordycipitaceae* also accounted for the total fungal community abundance.

The Venn diagram demonstrated that OTUs differed among the two moss species at different rocky desert types (Fig. 2c). The number of site-specific OTUs ranged from 115 (Hyo-Ve) to 321 (Hyp-Sl). In addition, a total of 245 OTUs were shared among all samples; these were defined as the core microorganisms of the fungal community. These OTUs were composed of a number of fungal groups, including *Ascomycota*, *Basidiomycota*, *Chytridiomycota*, and *Mucoromycota*.

Hierarchical cluster analysis revealed that the 18 fungal community samples that clustered into two groups corresponded very well with the two moss species (Fig. 2d). Clustering indicated that the structure of the fungal community was more closely related to bryophyte species rather than habitat. Therefore, the results suggest that moss species may be a major factor in changing the differentiation of microbial communities in karst rocky desertification ecosystems.

The LEfSe analysis revealed that 40 biomarkers affiliating with 4 phyla were sensitive to two moss species (*P* < 0.05, LDA > 2.0; Fig. 3b; Supplemental Table S6), which accounted for 4.5% of all taxa retrieved. For Hyp, two taxa within the classes *Pucciniomycetes* in *Basidiomycota* were more sensitive, while 11 *Agaricomycetes* within this order were significantly enriched in the Hyo species (Supplemental Fig. S7; Supplemental Table S6). In addition, the fungi (from phylum level to family level) that sensitive to three karst rocky desertification areas (slightly, severely, and very severely) of two moss species were analyzed (Supplemental Fig. S7).

### Microbial ecological function analysis

**Fig. 4:**
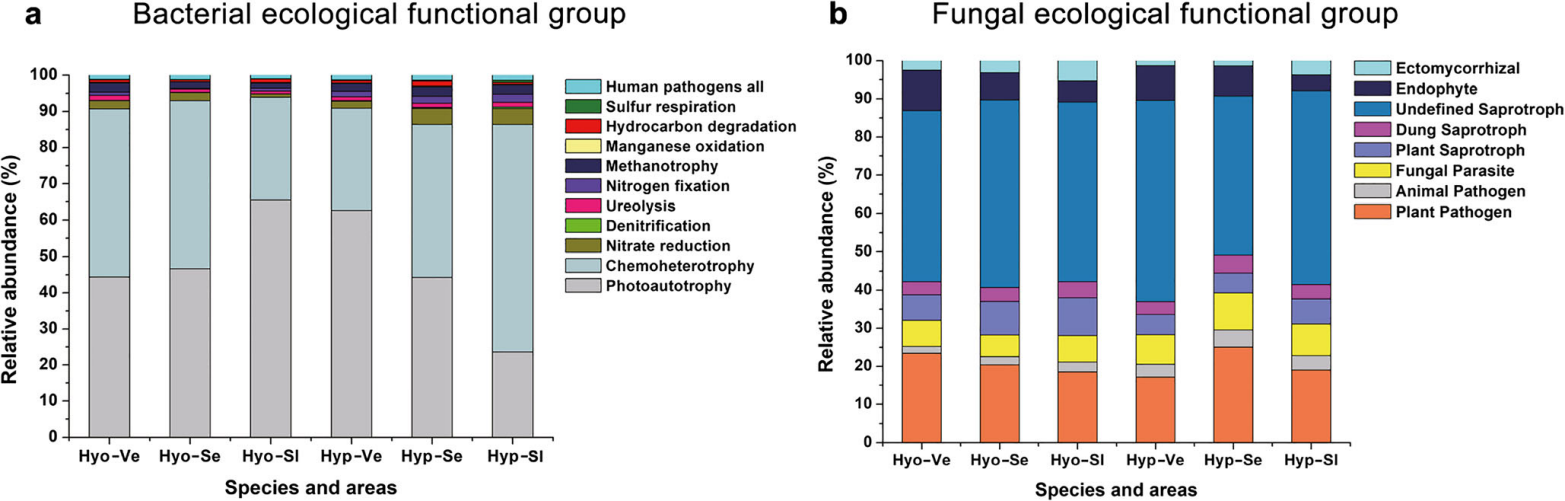
Changes in the ecological functional groups of the bacterial (**a**) and fungal (**b**) communities of two mosses at the OTU level. The two mosses including *H. involuta* and *H. leptothallum* were collected from very severely areas, severely areas, and slightly areas. In **a** and **b**, class names were color-coded on the right with the respective ecological function listed above

According to the results of species classification, the functional annotation of bacterial communities related to two mosses was conducted with the FAPROTAX database. Except for the unidentified groups, 39 functional groups of bacteria were obtained, and 11 functional groups with relatively high abundance are shown in Fig. 4a. The main functional groups in the two mosses, including photoautotrophic, chemoheterotrophic, nitrate reduction, methanotrophic, and human pathogens, and especially photoautotrophic and chemoheterotrophic groups, accounted for more than 85% of the total group. The proportion of dominant functional groups of the same moss bacterial community was not consistent in different karst rocky desertification areas. Similarly, the dominant functional groups of two different moss bacterial communities in the same karst rocky desertification area were also different in proportion, and there was no consistent change trend. Through comparative analysis of the FUNGuild database, except for the non-identified groups, the fungal communities of two mosses in different karst rocky desertification areas were divided into 8 ecological functional groups (Fig. 4b). Plant pathogen and saprotroph (plant saprotroph, dung saprotroph, and undefined saprotroph) were dominant groups in the two moss species, accounting for more than 70% of the total fungi. A small amount of animal pathogens and fungal parasites were observed. In particular, some entomopathogenic fungi related to *Cordyceps* were identified, such as *Hirsutella* and *Ophiocordyceps*, which have been reported to be symbiotic with plants and as a necessary link in the life cycle. In addition, some endophytes and ectomycorrhizae were identified.

### Co-occurrence patterns of bacteria and fungi

**Fig. 5:**
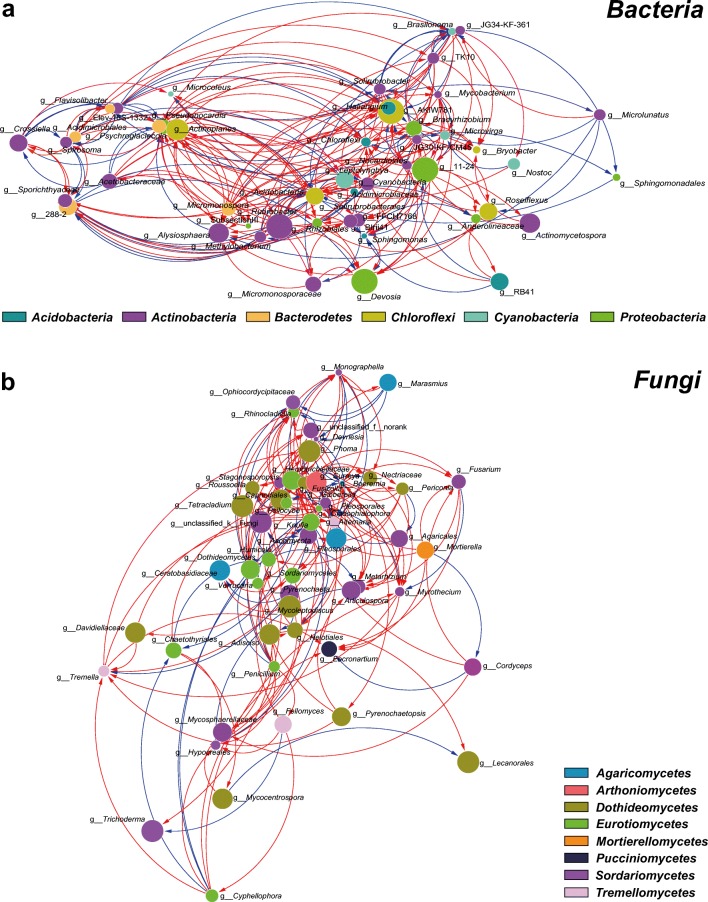
The networks visualize two moss species on the co-occurrence pattern between bacterial and fungal taxa at genus level. **a** The networks were constructed based on the top 50 bacterial genera of two moss species from all karst rocky desertification types. **b** The networks were constructed based on the top 50 fungal genera for all karst rocky desertification type. The node colors indicate that they belong to different phyla. The node size is proportional to the abundance of taxa, and the nodes filled with different colors indicate that they belong to different phyla. The edges are colored according to interaction types; positive correlations are labeled with red and negative correlations are colored in blue

In order to understand the correlation between species of moss-related microorganisms, we have selected the bacterial and fungal genera in the top 50 of the total horizontal abundance and calculated their Spearman rank correlation coefficient. Across all 18 samples from three karst rocky desertification types, correlation network analysis showed 137 strong positive correlations and 50 strong negative correlations among the top 50 dominant bacterial genera (*P* < 0.01, *R* > 0.6; Fig. 5a). The average path length of network was 2.248 edges with a diameter of 5 edges. The transitivity was 0.447 and the modularity index (MD) was 0.67, where MD > 0.5 suggests that the network has a modular structure. The co-occurring taxa mainly distributed in *Acidobacteria*, *Bacterodetes*, *Chloroflexi*, *Cyanobacteria*, and *Proteobacteria* phylum. When the distribution of nodes was modularized, all nodes were classified into two major modules (> 10 nodes). Based on betweenness centrality scores, the top three genera identified were *Solirubrobacter*, JG34-KF-361, and *Psychroglaciecola*, which indicates the critical roles these bacteria play as keystone taxa in the co-occurrence network.

The correlation network analysis of fungi showed 119 strong positive correlations and 48 strong negative correlations among the top 50 dominant genera (*P* < 0.01, *R* > 0.7; Fig. 5b). The average path length of network was 2.411 edges with a diameter of 5 edges. The MD was 0.58, where MD > 0.5 suggests that the network has a modular structure. However, there were no major modules when the distribution of nodes was modularized, and the top three genera identified were *Neofusicoccum*, *Cladosporium*, and *Mycosphaerella*, according to the betweenness centrality scores.

### The relationship between drought resistance properties of moss and moss microbial properties

To further characterize the differentiated effects of the karst rocky desertification type and moss species on the diversity of moss microbiomes, a permutation multivariate analysis of variance based on PERMANOVA was constructed (Table 4). Across all indexes, moss species (*P* = 0.001, *R*^2^ = 0.263) had the highest correlation with their symbiotic bacterial communities, followed by the rocky desertification type (*P* = 0.006, *R*^2^ = 0.198; Table 4). In addition, the drought resistance indexes SOD (*P* = 0.001, *R*^2^ = 0.164) and Pro (*P* = 0.002, *R*^2^ = 0.163) also had a great influence on the bacterial community. Hyp showed better drought resistance, with a mean SOD and Pro value of 292.03 and 1.42, respectively, whereas Hyo had a mean SOD and Pro value at 229.18 and 1.16, respectively (Supplemental Table S2). Similar to the bacterial communities, the fungal communities also exhibited a strong connection with moss species (*P* = 0.004, *R*^2^ = 0.204) and karst rocky desertification type (*P* = 0.017, *R*^2^ = 0.122), as well as the drought resistance indexes SOD (*P* = 0.017, *R*^2^ = 0.108) and Pro (*P* = 0.020, *R*^2^ = 0.109; Table 4).

Redundancy analysis and permutation test were used to determine the relationships among seven biochemical and environmental factors and bacterial and fungal community structures. Based on the detrended correspondence analysis (DCA) with species-sample data (97% similarity of samples from the OTU), if the size of lengths of the first axis were ≥ 3.5, CCA analysis is more accurate, and RDA is better than CCA if it is less than 3.5 (Shankar et al. 2017). From the RDA analysis of bacterial communities (axis lengths = 2.249), axis 1 explained 26.56% of the variance, and axis 2 explained another 8.67% (Supplemental Fig. S9a). The major biochemical factors driving moss bacterial community composition were POD (*P* = 0.001, *R*^2^ = 0.239), rain (*P* = 0.048, *R*^2^ = 0.235), Pro (*P* = 0.038, *R*^2^ = 0.157), and SOD (*P* = 0.012, *R*^2^ = 0.126). There was no significant correlation between other environmental factors and bacterial community structure (*P* > 0.05).

As the DCA results were less than 3.5, CCA analysis was carried out on the fungal community (lengths of gradient = 0.661). From the CCA analysis of the fungal communities (axis lengths = 2.249), axis 1 explained 11.55% of the variance, and axis 2 explained another 10.01% (Supplemental Fig. S9b). The major biochemical factors driving moss fungal community composition were TEM (*P* = 0.006, *R*^2^ = 0.293), POD (*P* = 0.036, *R*^2^ = 0.281), sun (*P* = 0.007, *R*^2^ = 0.264), SOD (*P* = 0.029, *R*^2^ = 0.152), Pro (*P* = 0.034, *R*^2^ = 0.136), and rain (*P* = 0.035, *R*^2^ = 0.125). Based on the above CCA/RDA analysis results, it revealed the influence of environmental factors and drought resistance indicators on the dominant microbial community, and the four variables POD, rain, Pro, and SOD have significant correlations with the microbial community.

**Fig. 6:**
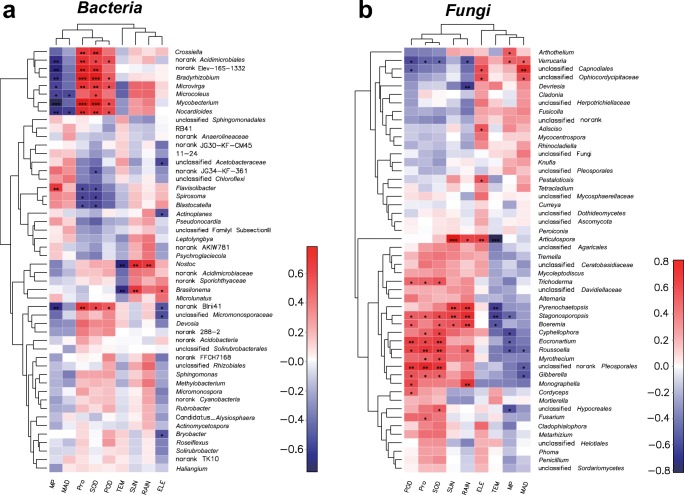
Association of microbiota with biochemical and environmental factors. Differentially abundant genera from the linear discriminant analysis were selected to calculate the Spearman’s correlation values for each factor: **a** bacteria and **b** fungi. “*,” “**,” and “***” represent 0.01 < *P* ≤ 0.05, 0.001 < *P* ≤ 0.01, and *P* ≤ 0.001, respectively

Finally, we further assessed the association between the specific genera in the microbial community and environmental variables with the Spearman correlation. From a systemic perspective, the genera with the top 50 abundance were selected for the analysis of both bacterial and fungal communities. Spearman’s correlation values were calculated for each genus compartment using the key microbiota that was differentially abundant according to the LEfSe analysis (Fig. 3). Figure 6 shows that there was a clear clustering on each correlation heatmap for the bacteria and fungi. According to Fig. 6 a, the environmental variables MP, MAD, Pro, SOD, and POD were clustered into one branch, which was a significant correlation with clade 1 of the bacterial genus. Among clade 1, *Bradyrhizobium*, *Mycobacterium*, *Crossiella*, *Microvirga*, and *Nocardioides* had a strong positive association with three drought resistance indexes: Pro, SOD, and POD, which were closely related to the drought adaptability of the mosses. *Bradyrhizobium* and *Microvirga* are known to be part of the rhizobia, which are gram-negative, nitrogen-fixing bacteria that form nodules on host plants. Rhizobia also have symbiotic relationships with legume plants, which cannot live without the essential nitrogen-fixing processes of these bacteria (Lodwig et al. 2003). The significant correlation between rhizobia and Pro or SOD indicated that moss-related microorganisms may help bryophyte drought resistance by linking these drought resistance indicators. MP, another key drought resistance factor along with moss that was negatively correlated with drought resistance of mosses, has a strong negative correlation with *Mycobacterium* and two other norank genera of *Acidimicrobiales*.

Interestingly, the top 50 genera of fungi were also grouped into 2 clades, and clade 2 was significantly positively correlated with the drought resistance indexes of Pro, POD, and SOD (Fig. 6b). However, the negative correlation values of the three indexes gradually changed to a positive value in clade 1 (Fig. 6b). Among them, some plant pathogens (*Phoma*, *Gibberella*, *Fusarium*, *Monographella*, *Penicillium*), endophytes (*Stagonosporopsis*), and insect pathogens (*Metarhizium*, *Cordyceps*) were positively correlated with the main drought resistance indexes. In addition, the *Articulospora* genus was significantly positively correlated with the three climate factors (sun, rain, and elevation) and negatively correlated with temperature. The results demonstrated that the system of drought resistance indexes of Pro, POD, and SOD and the combined microorganisms can be more helpful for bryophyte adaption to karst rocky desertification.

## Discussion

As a pioneer plant, mosses can grow well in harsh environments and play an irreplaceable role in soil and water conservation, plant succession, and environmental improvement in karst rocky desertification areas (Jia et al. 2014; Wang et al. 2015). Mosses are able to secrete an acidic substance that constantly dissolves the rock surface, along with their own organic matter that decomposes when the plant dies, creating a layer of fertile soil on the surface. This layer of soil, combined with the ability of the moss to retain water and soil, creates conditions for other plants to grow later on (Bates 2000; Glime 2007). In our survey, most of our sampling sites were stone surfaces without large shrubs or trees around them. Therefore, compared with other areas with abundant vegetation in karst topography, the analysis of dominant families and genera in serious rocky desertification habitat shows that the bryophytes are mainly temperately distributed species, and their species richness is relatively low (Wang 2011; Tan 2017; Luo 2017). However, most of these species showed strong drought resistance. Observation of external morphologies and determination of physiological indexes of resistance indicated that these mosses not only morphologically evolved the structure of adapting to the drought environment and reducing water loss but also physiologically formed many drought-tolerant mechanisms, improving their drought-tolerant abilities. These morphologies were observed in the following ways: (1) leaf morphology, with obvious concave or concave into a pocket, ovoid leaves, which are conducive to water storage [e.g., *H. leptothallum* (Supplemental Fig. S2), *T. crispulum*, *B. argenteum*]; (2) leaf color, the color of plant body is mostly gray-green, yellow-green, or yellow-brown, and the top of stems are light green, which could reflect more light, reduce leaf temperature and water loss; (3) the warty structure inside the leaf [e.g., *H. involuta* (Supplemental Fig. S3), *D. fallax*, *T. crispulum*, *T. kanedae*, *D. constrictus* var. *constrictus*], which helps to reflect sunlight and reduces transpiration; and (4) plant height and rhizoid adhesion, for the erect species, the plant is generally less than 1 cm [e.g., *H. involuta* (Supplemental Fig. S3), *H. javanica*, *B. argenteum*], and the creeping species has a well-developed rhizoid [e.g., *H. leptothallum* (Supplemental Fig. S2), *P. euchloron*], allowing them to survive on exposed rock. In this study, it was found that most bryophytes adapted to rocky desertification adopted asexual reproduction, which was consistent with the study of Newton and Mishler (1994), and they noted that mosses mainly produced vegetative propagation under adverse conditions. Therefore, ecologically, the way in which these mosses reproduce asexually through a large number of gametophyte fragments or gemma (Supplemental Fig. S3) is a concrete manifestation of their R-type ecological strategy to compensate for the environmental impact on the survival of the population with a high reproductive rate (Li et al. 2008).

According to previous reports (Liu et al. 2014; Opelt and Berg 2004; Spiess et al. 1986; Tang et al. 2016), there are many microorganisms inhabited in or attached on the surface of mosses, which might play an important role in enhancing the environmental adaptability of their hosts by promoting their growth or improving their defense ability. Our results provide some important information on the correlations between moss species and microbes. Overall, compared with the rocky desertification habitat, bryophyte species have a profound impact on the structure of bacterial and fungal communities depending on the clustering and LEfSe results (Figs. 2 and 3). In addition, the composition of bacteria and fungi on mosses in different classes of rocky desertification areas was not completely consistent. *Actinobacteria*, *Proteobacteria*, *Cyanobacteria*, *Chloroflexi*, and *Acidobacteria* were the dominant phyla in all samples. Consistent with other reports, the members of the phyla *Proteobacteria*, *Actinobacteria*, and *Acidobacteria* were also detected as the most dominant bacteria in many moss hosts, but the proportion of these phyla were differentially varied across the different hosts (Tang et al. 2016; Koua et al. 2015; Faria et al. 2013). Remarkably, at the phylum level, a significant proportion of bacteria were *Cyanobacteria*, especially the proportion of cyanobacteria in the very severely area of Hyp species, which was up to 30.24%. There was a close correlation between moss and cyanobacteria. Previous studies have shown that cyanobacteria are considered the main N_2_-fixing bacteria in forest and desert mosses, and moss-associated cyanobacteria are a major source of new N to the boreal forest and arid lands (Yeager et al. 2004, 2007; Gavazov et al. 2010; Sorensen and Michelsen 2011; Stewart et al. 2011; Zhang et al. 2011; Lett and Michelsen 2014). Many studies have reported that the microbes that coexist with moss contain a variety of N_2_-fixing cyanobacteria, including *Cratoneuron filicinum*, *Pylaisiella polyantha*, and *Campyliadelphus polygamum* (Ma et al. 2017). Our data also showed that two dominant mosses are colonized by a variety of bacterial groups that may contribute to N_2_ fixation, especially heterotrophs and methane-oxidizing bacteria (methanotrophs) of the class *Alphaproteobacteria* (Putkinen et al. 2012; Bragina et al. 2013). Thus, these symbiotic microbes may potentially provide organic matter and oxygen to their hosts via biological nitrogen fixation and photosynthesis.

Combined analyses of the bacteria and fungi associated with two mosses from three karst rocky desertification areas via NMDS patterns, hierarchical clustering tree, and PERMANOVA (Figs. 1b, 2b, d; Table 4) showed that samples from the same moss generally displayed more similar bacterial communities, although the microbial community associated with the moss from Hyp-Se-3 displayed more scattered distribution. Thus, we inferred that the microbial community structure of moss under a rocky desertification habitat was primarily predictive of the moss species itself. Our results were different from previous studies, which noted that the main factor affecting the structure of bacterial communities was the sampling site. The possible reason for this result is that the two dominant mosses have different adaptation mechanisms under the extreme habitat of rocky desertification. *H. leptothallum* is densely creeping and forming large communities with the largest frequency and coverage; nevertheless, *H. involuta* is densely clustering and forming small patches on exposed rock surfaces, and moreover, there were significant differences in drought tolerance morphology and drought tolerance indexes between the above two species. Each moss species represented a specific environmental niche, and the structural composition was closely related to microbial community function; therefore, the niche was considered to be a determining factor of bacterial community in the mosses to some extent. In karst habitats, coexisting microorganisms play an important role in early vegetation succession and late vegetation succession (Kardol et al. 2007). According to our results, three drought resistance indexes, Pro content, SOD and POD activity, which mainly reflect the adaptability of plants to adversity, have a strong correlation with a variety of functional microbes, whether bacteria or fungi, such as some rhizobia, photoautotrophic bacteria, and endosymbiont fungi. These significant correlations further suggested that moss-related microbes play an irreplaceable role in adaptation to the arid environment of dominated mosses in karst rocky desertification habitats.

The prediction of the ecological function of bacteria and fungi further confirmed the positive role of the microbial community in moss environmental adaptation. In the bacterial community, photoautotrophic and chemoheterotrophic groups accounted for 85% of the total group, mainly including *Bradyrhizobium*, *Herbaspirillum*, *Cellulomonas*, *Blastococcus*, etc. A high abundance of photoautotrophic and chemoheterotrophic bacteria could contribute to the acquisition of carbon and nitrogen sources and the degradation of organic matter for moss hosts. Moreover, a high proportion of nitrate and nitrite reduction groups were mainly in the genus level of *Steroidobacter*, *Georgfuchsia*, and others, indicating that bacteria played an important role in increasing available nitrogen nutrients in moss. It was also noted that two bacterial genera were found to undergo manganese oxidation, and they are often used to monitor air and soil pollution (Wang et al. 2015). Among fungal communities, the plant pathogen and saprotroph (plant saprotroph, dung saprotroph, and undefined saprotroph) were the most dominant groups, and some endophytes and ectomycorrhizae were identified, indicating that the fungi could obtain nutrition mainly by destroying dead host cells or damaging host cells in moss and could also exchange resources with host cells for nutrients through endophytic fungi, which may in turn promote moss growth. A small amount of animal pathogens and fungal parasites were observed. In particular, some entomopathogenic fungi related to *Cordyceps* have been identified, such as *Hirsutella* and *Ophiocordyceps*, which have been reported to be symbiotic with plants and a necessary link in the life cycle. Most *Cordyceps* species are found in forests, river valleys, and alpine regions, which require high air humidity (Qu et al. 2018). However, *Cordyceps* species have not been reported in karst rocky desertification arid regions. Our findings broaden the understanding of these fungi, which could also exist in arid environments, coexisting with plants and taking chances to infect insects.

With excellent drought resistance qualities, mosses have the potential to be an excellent karst rocky desertification area restoration material. Our results suggest that *H. leptothallum* and *H. involuta* could be ideal candidates for moss biocrust cultivation for restoration purposes. With optimization, these mosses and growth methods are the key step in developing moss-based biocrust rehabilitation technology.

Taken together, we have comprehensively investigated the biodiversity of crust bryophytes in karst rocky desertification areas of Guizhou Province and found that dominant bryophytes exhibit specific drought resistance from morphology and physiology. In addition, we also showed a close correlation between two dominant moss species and the function, diversity, and ecological adaption ability of microbes associated with three karst rocky desertification areas grown in Guizhou Province. While the rocky desertification type also altered the bacterial and fungal community composition and diversity, the moss species much more strongly affected the diversity and changed the community composition of these microbial groups. Several bacterial and fungal taxa were identified as potential keystone microbiome taxa, which might control the microbiome in functioning to help bryophytes better adapt to the environment and occupy ecological niches. This finding is of far-reaching importance for understanding microbiome communities and karst rocky desertification system stability. Future subsequent vegetation restoration and environmental improvement studies in karst rocky desertification in general should include moss-related microorganism community analyses, as otherwise important information on microbiome structures might be missing. Overall, we propose that this survey could provide important information on the better use of mosses as environmental improvers in the future.

## Electronic supplementary material

ESM 1 (PDF 3588 kb)
