# Supplementary material for: Bryophytes and the symbiotic microorganisms, the pioneers of vegetation restoration in karst rocky desertification areas in southwestern China
Source: Appl Microbiol Biotechnol. 2019 Dec 10;104(2):873–91. doi: 10.1007/s00253-019-10235-0 (PMC6943408; doi:10.1007/s00253-019-10235-0)
Supplement: Supplementary file 1 — (PDF 3588 kb) [file 253_2019_10235_MOESM1_ESM.pdf]

Applied Microbiology and Biotechnology

Bryophytes and the symbiotic microorganisms, the pioneers of vegetation restoration in karst rocky desertification areas in southwestern China

Wei Cao[1,2&], Yuanxin Xiong[1&], Degang Zhao[2], Hongying Tan[3], Jiaojiao Qu[4*]

[*] Jiaojiao Qu

aileenqu028@outlook.com

[1] College of Life Sciences, Guizhou University, Guiyang, 550025, China.

[2] The Key Laboratory of Plant Resources Conservation and Germplasm Innovation in Mountainous Region (Ministry of Education), Institute of Agro-Bioengineering and College of Life Sciences, Guizhou University, Guiyang, 550025, China.

[3] Guiyang A'ha Lake National Wetland Park Management Division, Guiyang, 550002, China.

[4] College of Tea Sciences, Guizhou University, Guiyang, 550025, China.

[&] These authors contributed equally to this work and should be considered co-first authors

Supplemental Table S1

Statistics of moss specimens collected from all sample sites

Sample plot	Family	Species	Frequency
Hongguo town, Panzhou City; E107°26'50", N25°42'57"; 1700-1800m; 7 quadrats	*Ptychomitriaceae* Schimp.	*Ptychomitrium gardneri* Lesq.	1
	Leucobryaceae Schimp.	*Campylopus umbellatus* (Arnott) Paris	3
	Pottiaceae Schimp.	*Didymodon fallax* (Hedw.) R. H. Zander	1
		Didymodon ferrugineus (Schimp. ex Besch.) Hill	1
		Didymodon vinealis (Brid.) R. H. Zander	1
		Didymodon constrictus var. *constrictus* (Mitt.) Saito	1
		Hyophila javanica (Nees & Blume) Brid.	2
		Hyophila involuta (Hook.) A. Jaeger	2
		Pseudosymblepharis angustata (Mitt.) Hilp.	2
		Trichostomum crispulum Bruch	1
		Trichostomum planifolium (Dixon) R. H. Zander	1
	Bryaceae Schwägr.	*Brachymenium systylium* (C.Muell.) Jaegr.	1
		Bryum algovicum Sendt.	2
		Bryum argenteum Hedw.	1
	Thuidiaceae Schimp.	*Thuidium cymbifolium* (Dozy & Molk.) Dozy & Molk.	1
		Thuidium kanedae Sakurai	1
	Brachytheciaceae Schimp.	*Brachythecium perminusculum* Müll. Hal.	1
		Brachythecium pulchellum Broth. & Paris	1
		Eurhynchium eustegium (Besch.) Dixon	2
	Meteoriaceae Kindb.	*Meteorium papillarioides* Nog.	1
		Meteorium polytrichum Dozy & Molk.	1
	Hypnaceae Schimp.	*Hypnum calcicola* Ando	
		Hypnum leptothallum (Müll. Hal.) Paris	
Houchang town, Shuicheng County; E104°42'31", N26°43'29"; 1850-2000m; 6 quadratss	*Ptychomitriaceae* Schimp.	*Ptychomitrium gardneri* Lesq.	2
	Pottiaceae Schimp.	*Didymodon fallax* (Hedw.) R. H. Zander	1
		Hyophila involuta (Hook.) A. Jaeger	1
		Hyophila javanica (Nees & Blume) Brid.	2
		Pseudosymblepharis angustata (Mitt.) Hilp.	1
		Trichostomum crispulum Bruch	1
		Trichostomum planifolium (Dixon) R. H. Zander	1
	Bryaceae Schwägr.	*Brachymenium leptophyllum* (Müll. Hal.) A. Jaeger	1
		Brachymenium longidens Renauld & Cardot	2
		Bryum algovicum Sendt.	1
		Bryum argenteum Hedw.	1
		Plagiobryum demissum (Hook.) Lindb.	1

	Thuidiaceae Schimp.	*Thuidium cymbifolium* (Dozy & Molk.) Dozy & Molk.	2
		Thuidium kanedae Sakurai	3
	Brachytheciaceae Schimp.	*Palamocladium euchloron* (Müll. Hal.) Wijk & Margad.	1
	Hypnaceae Schimp.	*Gollania cylindricarpa* (Mitt.) Broth.	2
		Hypnum leptothallum (Müll. Hal.) Paris	5
Baoji Village, Panzhou City; E104°53'04", N25°54'57"; 1750-1900m; 26 quadrats	*Ptychomitriaceae* Schimp.	*Ptychomitrium gardneri* Lesq.	6
	Leucobryaceae Schimp.	*Campylopus umbellatus* (Arnott) Paris	1
	Pottiaceae Schimp.	*Didymodon constrictus* var. *constrictus* (Mitt.) Saito	1
		Didymodon ditrichoides (Broth.) X. J. Li & S. He	1
		Didymodon fallax (Hedw.) R. H. Zander	8
		Hyophila involuta (Hook.) A. Jaeger	1
		Hyophila javanica (Nees & Blume) Brid.	1
		Pseudosymblepharis angustata (Mitt.) Hilp.	1
		Pseudosymblepharis duriuscula (Mitt.) P. C. Chen	1
		Trichostomum crispulum Bruch	2
		Weissia breviseta (Thér.) P. C. Chen	1
	Bryaceae Schwägr.	*Brachymenium nepalense* Hook.	1
		Bryum algovicum Sendt.	2
		Bryum apiculatum Schwaegr.	1
		Bryum argenteum Hedw.	7
		Bryum caespiticium Hedw.	1
		Bryum calophyllum R. Br.	1
		Bryum dichotomum Hedw.	1
		Bryum funkii Schwägr.	1
		Bryum recurvulum Mitt.	1
		Bryum tuberosum Mohamed & Damanhuri	1
		Bryum yuennanense Broth.	2
		Plagiobryum demissum (Hook.) Lindb.	3
	Mniaceae Schwägr.	*Plagiomnium cuspidatum* (Hedw.) T. J. Kop.	2
		Plagiomnium integrum (Bosch & Sande Lac.) T. J. Kop.	3
		Plagiomnium rostratum (Schrad.) T. J. Kop.	1
	Leskeaceae Schimp.	*Lindbergia serrulatus* C. Gao	1
	Thuidiaceae Schimp.	*Haplocladium angustifolium* (Hampe & Müll. Hal.) Broth.	1
		Thuidium glaucinoides Broth.	1
		Thuidium kanedae Sakurai	11
		Thuidium subglaucinum Cardot	1
	Brachytheciaceae Schimp.	*Brachythecium amnicola* Müll. Hal.	1
		Brachythecium brotheri Paris	1
		Brachythecium campylothallum Müll. Hal.	1
		Brachythecium fasciculirameum Müll. Hal.	1

		Brachythecium piligerum Cardot	2
		Brachythecium plumosum (Hedw.) Bruch & Schimp.	2
		Brachythecium viridefactum Müll. Hal.	1
		Eurhynchium eustegium (Besch.) Dixon	3
		Eurhynchium longirameum (Müll. Hal.) Y. F. Wang & R. L. Hu	2
		Homalothecium leucodonticaule (Müll. Hal.) Broth.	2
		Palamocladium euchloron (Müll. Hal.) Wijk & Margad.	9
		Palamocladium leskeoides (Hook.) E. Britton	1
		Rhynchostegium pallenticaule Müll. Hal.	1
		Rhynchostegium serpenticaule (Müll. Hal.) Broth.	2
	Meteoriaceae Kindb.	*Meteoriopsis reclinate* (Müll. Hal.) M. Fleisch.	3
		Meteorium polytrichum Dozy & Molk.	2
	Hypnaceae Schimp.	*Hypnum calcicola* Ando	1
		Hypnum leptothallum (Müll. Hal.) Paris	5
		Hypnum oldhamii (Mitt.) A. Jaeger	1
	Pylaisiaceae Schimp.	*Pylaisia extenta* (Mitt.) A. Jaeger	1
		Pylaisia polyantha (Hedw.) Bruch & Schimp.	1
	Anomodontaceae Kindb.	*Anomodon viticulosus* (Hedw.) Hook. & Taylor	6
		Anomodon minor (Hedw.) Lindb.	2
Longguang Town, Xingyi City; E105°06'30", N25°04'12"; 1400-1500m; 9 quadrats	*Fissidentaceae* Schimp.	*Fissidens nobilis* Griff.	1
	Pottiaceae Schimp.	*Barbula amplexifolia* (Mitt.) A. Jaeger	1
		Didymodon fallax (Hedw.) R. H. Zander	1
		Didymodon vinealis (Brid.) R. H. Zander	1
		Hyophila involuta (Hook.) A. Jaeger	1
		Hyophila javanica (Nees & Blume) Brid.	7
		Pseudosymblepharis angustata (Mitt.) Hilp.	1
		Trichostomum crispulum Bruch	4
		Trichostomum planifolium (Dixon) R. H. Zander	2
		Weissia breviseta (Thér.) P. C. Chen	2
	Racopilaceae Kindb.	*Racopilum cuspidigerum* (Schwägr.) Ångström	1
	Bryaceae Schwägr.	*Brachymenium longidens* Renauld & Cardot	1
		Bryum pachytheca Müll. Hal.	1
Zerong Village, Xingyi City; E104°54'40", N24°59'57"; 1000-1300m; 18 quadrats	*Ptychomitriaceae* Schimp.	*Ptychomitrium gardneri* Lesq.	2
	Pottiaceae Schimp.	*Barbula sordida* Besch.	1
		Barbula unguiculata Hedw.	2
		Didymodon fallax (Hedw.) R. H. Zander	2
		Hyophila involuta (Hook.) A. Jaeger	4
		Tortella tortuosa (Hedw.) Limpr.	1

		Trichostomum brachydontium Bruch	2
		Trichostomum crispulum Bruch	1
		Trichostomum platyphyllum (Iisiba) P. C. Chen	4
		Trichostomum sinochenii Redf. & B. C. Tan	1
		Trichostomum tenuirostre (Hook. f. & Taylor) Lindb.	1
		Weissia brachycarpa (Nees & Hornsch.) Jur.	1
		Weissia controversa Hedw.	1
		Weissia edentula Mitt.	1
		Weissia longifolia Mitt.	1
	Bartramiaceae Schwägr.	*Philonotis turneriana* (Schwägr.) Mitt.	1
	Bryaceae Schwägr.	*Brachymenium leptophyllum* (Müll. Hal.) A. Jaeger	1
		Brachymenium longidens Renauld & Cardot	2
		Bryum argenteum Hedw.	1
		Bryum caespiticium Hedw.	1
		Bryum coronatum Schwägr.	1
		Bryum pallens Sw.	1
		Bryum pallescens var. *pallescens* Schleich.	1
		Bryum uliginosum (Brid.) Bruch & Schimp.	2
		Plagiobryum demissum (Hook.) Lindb.	1
	Mniaceae Schwägr.	*Plagiomnium ellipticum* (Brid.) T. J. Kop.	2
	Racopilaceae Kindb.	*Racopilum cuspidigerum* (Schwägr.) Ångström	2
	Brachytheciaceae Schimp.	*Brachythecium piligerum* Cardot	1
		Eurhynchium eustegium (Besch.) Dixon	2
		Eurhynchium longirameum (Müll. Hal.) Y. F. Wang & R. L. Hu	1
		Rhynchostegium serpenticaule (Müll. Hal.) Broth.	2
	Hypnaceae Schimp.	*Hypnum leptothallum* (Müll. Hal.) Paris	11
	Meteoriaceae Kindb.	*Aerobryopsis subdivergens* (Broth.) Broth.	1
		Meteorium papillarioides Nog.	1
		Meteorium polytrichum Dozy & Molk.	1
	Entodontaceae Kindb.	*Entodon viridulus* Cardot	1
	Anomodontaceae Kindb.	*Anomodon minor* (Hedw.) Lindb.	5
		Anomodon viticulosus (Hedw.) Hook. & Taylor	1
	Aytoniaceae Cavers	*Asterella yoshinagana* (Horik.) Horik.	1
	Porellaceae Cavers	*Porella chinensis* (Steph.) S. Hatt.	1
Mashan Town, Wangmo County; E106°20'46", N25°09'48"; 800-900m; 17 quadrats	*Ptychomitriaceae* Schimp.	*Ptychomitrium gardneri* Lesq.	1
	Pottiaceae Schimp.	*Barbula unguiculata* Hedw.	2
		Didymodon constrictus var. *constrictus* (Mitt.) Saito	6
		Didymodon ferrugineus (Schimp. ex Besch.) Hill	1
		Didymodon vinealis (Brid.) R. H. Zander	1
		Hyophila involuta (Hook.) A. Jaeger	1

		Pseudosymblepharis duriuscula (Mitt.) P. C. Chen	1
		Trichostomum planifolium (Dixon) R. H. Zander	1
	Bryaceae Schwägr.	*Anomobryum gemmigerum* Broth.	1
		Bryum alpinum Huds ex With.	1
	Mniaceae Schwägr.	*Plagiomnium cuspidatum* (Hedw.) T. J. Kop.	6
		Plagiomnium maximoviczii (Lindb.) T. J. Kop.	1
		Plagiomnium vesicatum(Besch.) T. J. Kop.	1
	Orthotrichaceae Arn.	*Macromitrium gymnostomum* Sull. & Lesq.	1
	Racopilaceae Kindb.	*Racopilum cuspidigerum* (Schwägr.) Ångström	4
	Thuidiaceae Schimp.	*Haplocladium angustifolium* (Hampe & Müll. Hal.) Broth.	2
		Haplocladium microphyllum (Hedw.) Broth.	1
		Thuidium kanedae Sakurai	4
	Brachytheciaceae Schimp.	*Brachythecium fasciculirameum* Müll. Hal.	1
		Brachythecium helminthocladum Broth. & Paris	1
		Brachythecium pulchellum Broth. & Paris	1
		Bryhnia brachycladula Cardot	1
		Palamocladium euchloron (Müll. Hal.) Wijk & Margad.	3
	Meteoriaceae Kindb.	*Meteorium polytrichum* Dozy & Molk.	2
	Pylaisiaceae Schimp.	*Homomallium yuennanense* Broth.	1
	Sematophyllaceae Broth.	*Trichosteleum lutschianum* (Broth. & Paris) Broth.	1
	Entodontaceae Kindb.	*Entodon viridulus* Cardot	1
		Entodon yunnanensis Thér.	1
	Neckeraceae Schimp.	*Neckera pennata* Hedw.	1
		Neckera yezoana Besch.	1
	Anomodontaceae Kindb.	*Anomodon minor* (Hedw.) Lindb.	1
		Herpetineuron toccoae (Sull. & Lesq.) Cardot	1
	Aytoniaceae Cavers	*Asterella mitsumiensis* Schimizu & S. Hatt.	1
	Porellaceae Cavers	*Porella densifolia* subsp. *appendiculata* (Steph.) S. Hatt.	1
		Porella densifolia var. *densifolia* (Steph.) S. Hatt.	1
		Porella obtusata (Taylor) Trevis.	1
Qianxi Village, Bijie City; E105°10'39", N27°10'37"; 1600-1700m; 15 quadrats	*Ptychomitriaceae* Schimp.	*Ptychomitrium gardneri* Lesq.	1
	Grimmiaceae Arn.	*Schistidium apocarpum* (Hedw.) Bruch & Schimp.	2
	Dicranaceae Schimp.	*Dicranum leiodontum* Cardot	1
	Leucobryaceae Schimp.	*Campylopus umbellatus* (Arnott) Paris	1
	Pottiaceae Schimp.	*Didymodon constrictus* var. *flexicuspis* (P. C. Chen) Saito	2
		Didymodon ditrichoides (Broth.) X. J. Li & S. He	2
		Didymodon fallax (Hedw.) R. H. Zander	5
		Didymodon ferrugineus (Schimp. ex Besch.) Hill	2
		Hyophila involuta (Hook.) A. Jaeger	3

		Hyophila javanica (Nees & Blume) Brid.	2
		Weissia breviseta (Thér.) P. C. Chen	1
	Bryaceae Schwägr.	*Bryum argenteum* Hedw.	1
		Bryum caespiticium Hedw.	2
		Bryum calophyllum R. Br.	1
		Bryum dichotomum Hedw.	1
		Bryum funkii Schwägr.	2
		Bryum pallescens var. *pallescens* Schleich.	1
		Bryum recurvulum Mitt.	4
	Mniaceae Schwägr.	*Plagiomnium cuspidatum* (Hedw.) T. J. Kop.	2
	Orthotrichaceae Arn.	*Orthotrichum anomalum* Hedw.	2
	Thuidiaceae Schimp.	*Thuidium cymbifolium* (Dozy & Molk.) Dozy & Molk.	1
		Thuidium kanedae Sakurai	3
	Brachytheciaceae Schimp.	*Palamocladium euchloron* (Müll. Hal.) Wijk & Margad.	2
	Hypnaceae Schimp.	*Gollania clarescens* (Mitt.) Broth.	2
		Gollania schensiana Dixon ex Higuchi	1
	Aytoniaceae Cavers	*Reboulia hemisphaerica* (L.) Raddi	1
	Porellaceae Cavers	*Porella obtusata* (Taylor) Trevis.	1
Sanglang Town, Wangmo County; E106°26'59", N25°14'28"; 950-1100m; 10 quadrats	*Pottiaceae* Schimp.	*Barbula unguiculata* Hedw.	1
		Didymodon constrictus var. *constrictus* (Mitt.) Saito	1
		Didymodon fallax (Hedw.) R. H. Zander	1
		Hyophila javanica (Nees & Blume) Brid.	3
		Trichostomum crispulum Bruch	4
		Trichostomum platyphyllum (Iisiba) P. C. Chen	1
		Weissia breviseta (Thér.) P. C. Chen	1
	Mniaceae Schwägr.	*Plagiomnium cuspidatum* (Hedw.) T. J. Kop.	2
		Plagiomnium integrum (Bosch & Sande Lac.) T. J. Kop.	1
	Racopilaceae Kindb.	*Racopilum cuspidigerum* (Schwägr.) Ångström	3
	Brachytheciaceae Schimp.	*Eurhynchium eustegium* (Besch.) Dixon	1
		Okamuraea hakoniensis (Mitt.) Broth.	3
	Meteoriaceae Kindb.	*Meteorium polytrichum* Dozy & Molk.	2
	Hypnaceae Schimp.	*Hypnum leptothallum* (Müll. Hal.) Paris	1
	Entodontaceae Kindb.	*Entodon sullivantii* (Müll. Hal.) Lindb. var. *versicolor* (Besch.) Mizush	1
	Anomodontaceae Kindb.	*Anomodon viticulosus* (Hedw.) Hook. & Taylor	4
		Herpetineuron toccoae (Sull. & Lesq.) Cardot	1
	Porellaceae Cavers	*Porella handelii* S. Hatt.	4
	Lejeuneaceae Casares-Gil.	*Trocholejeunea sandvicensis* (Gottsche) Mizt.	1

Huajiang Town, Guanling County; E105°34'01", N25°43'06"; 750-900m; 16 quadrats	*Pottiaceae* Schimp.	*Barbula javanica* Dozy & Molk.	1
		Barbula javanica Dozy & Molk.	3
		Chenia leptophylla (Müll. Hal.) R. H. Zander	2
		Didymodon constrictus var. *constrictus* (Mitt.) Saito	1
		Didymodon fallax (Hedw.) R. H. Zander	5
		Hyophila involuta (Hook.) A. Jaeger	5
		Pseudosymblepharis angustata (Mitt.) Hilp.	1
		Pseudosymblepharis angustata (Mitt.) Hilp.	1
	Bryaceae Schwägr.	*Bryum algovicum* Sendt.	1
		Bryum alpinum Huds ex With.	1
		Bryum argenteum Hedw.	4
		Bryum capillare Hedw.	1
		Bryum dichotomum Hedw.	1
		Bryum funkii Schwägr.	4
		Bryum pachytheca Müll. Hal.	2
		Bryum recurvulum Mitt.	6
		Bryum yuennanense Broth.	1
	Brachytheciaceae Schimp.	*Brachythecium planiusculum* Müll. Hal.	1
		Brachythecium viridefactum Müll. Hal.	2
		Okamuraea hakoniensis (Mitt.) Broth.	5
		Palamocladium euchloron (Müll. Hal.) Wijk & Margad.	2
	Meteoriaceae Kindb.	*Meteorium polytrichum* Dozy & Molk.	4
	Hypnaceae Schimp.	*Gollania clarescens* (Mitt.) Broth.	1
		Hypnum leptothallum (Müll. Hal.) Paris	15
		Taxiphyllum taxirameum (Mitt.) M. Fleisch.	1
	Entodontaceae Kindb.	*Erythrodontrium julaceum* (Schwägr.) Paris	1
Xiayun Town, Anshun City; E106°17'49", N26°26'57"; 1200-1300m; 4 quadrats	*Grimmiaceae* Arn.	*Niphotrichum japonicum* (Dozy & Molk.) Bednarek-Ochyra & Ochyra	1
	Pottiaceae Schimp.	*Barbula unguiculata* Hedw.	2
		Didymodon fallax (Hedw.) R. H. Zander	2
		Trichostomum crispulum Bruch	2
		Weissia breviseta (Thér.) P. C. Chen	1
	Bryaceae Schwägr.	*Bryum argenteum* Hedw.	1
	Hypnaceae Schimp.	*Hypnum calcicola* Ando	1
		Hypnum leptothallum (Müll. Hal.) Paris	4

Zhongjie Village, Yanhe County; E108°32'47"; N28°29'12"; 500-600m; 17 quadrats	*Pottiaceae* Schimp.	*Chenia leptophylla* (Müll. Hal.) R. H. Zander	2
		Didymodon constrictus var. *flexicuspis* (P. C. Chen) Saito	2
		Hyophila involuta (Hook.) A. Jaeger	1
		Trichostomum crispulum Bruch	3
	Bryaceae Schwägr.	*Bryum algovicum* Sendt.	1
		Bryum argenteum Hedw.	2
		Bryum caespiticium Hedw.	1
		Bryum coronatum Schwägr.	1
		Bryum dichotomum Hedw.	2
		Bryum lonchocaulon Müll. Hal.	1
		Bryum pallescens var. *pallescens* Schleich.	1
		Bryum pallescens var. *subrotundum* (Brid.) Bruch & Schimp	1
		Bryum recurvulum Mitt.	5
		Plagiobryum demissum (Hook.) Lindb.	1
	Brachytheciaceae Schimp.	*Brachythecium dicranoides* Müll. Hal.	1
		Brachythecium perminusculum Müll. Hal.	1
		Brachythecium planiusculum Müll. Hal.	1
		Brachythecium plumosum (Hedw.) Bruch & Schimp.	1
		Eurhynchium laxirete Broth.	1
		Palamocladium euchloron (Müll. Hal.) Wijk & Margad.	1
		Rhynchostegium subspeciosum (Müll. Hal.) Müll. Hal.	1
	Hypnaceae Schimp.	*Hypnum leptothallum* (Müll. Hal.) Paris	15
	Entodontaceae Kindb.	*Entodon viridulus* Cardot	1
Shazi Town, Yanhe County; E108°32'16", N28°33'36"; 600-700m; 5 quadrats	*Pottiaceae* Schimp.	*Barbula javanica* Dozy & Molk.	1
		Barbula unguiculata Hedw.	3
		Didymodon constrictus var. *constrictus* (Mitt.) Saito	1
		Didymodon constrictus var. *flexicuspis* (P. C. Chen) Saito	1
		Didymodon fallax (Hedw.) R. H. Zander	2
		Didymodon ferrugineus (Schimp. ex Besch.) Hill	1
		Hyophila involuta (Hook.) A. Jaeger	1
		Trichostomum crispulum Bruch	2
		Weissia longifolia Mitt.	1
	Bryaceae Schwägr.	*Bryum blindii* Bruch & Schimp.	1
		Bryum funkii Schwägr.	1
	Thuidiaceae Schimp.	*Thuidium kanedae* Sakurai	2
	Brachytheciaceae Schimp.	*Rhynchostegium fauriei* Cardot	1
	Hypnaceae Schimp.	*Gollania clarescens* (Mitt.) Broth.	3

		Hypnum leptothallum (Müll. Hal.) Paris	2
Qiantao Village, Guiyang City; E106°47'53"; N26°20'46"; 1250-1450m; 18 quadrats	*Ptychomitriaceae* Schimp.	*Ptychomitrium gardneri* Lesq.	1
	Leucobryaceae Schimp.	*Campylopus umbellatus* (Arnott) Paris	1
	Pottiaceae Schimp.	*Anoectangium thomsonii* Mitt.	1
		Barbula javanica Dozy & Molk.	2
		Barbula sordida Besch.	2
		Barbula unguiculata Hedw.	3
		Didymodon constrictus var. *constrictus* (Mitt.) Saito	4
		Didymodon constrictus var. *flexicuspis* (P. C. Chen) Saito	3
		Didymodon ditrichoides (Broth.) X. J. Li & S. He	1
		Didymodon fallax (Hedw.) R. H. Zander	6
		Hyophila involuta (Hook.) A. Jaeger	4
		Hyophila javanica (Nees & Blume) Brid.	2
		Trichostomum crispulum Bruch	10
		Trichostomum zanderi Redf. & B. C. Tan	1
		Weissia breviseta (Thér.) P. C. Chen	4
	Bryaceae Schwägr.	*Anomobryum gemmigerum* Broth.	2
		Brachymenium exile (Dozy &. Molk.) Bosch & Sande Lac.	1
		Bryum alpinum Huds ex With.	2
		Bryum argenteum Hedw.	3
		Bryum caespiticium Hedw.	1
		Bryum coronatum Schwägr.	1
		Bryum dichotomum Hedw.	1
		Bryum funkii Schwägr.	1
		Bryum pachytheca Müll. Hal.	1
		Bryum pallescens var. *pallescens* Schleich.	2
		Bryum recurvulum Mitt.	6
		Bryum yuennanense Broth.	1
	Thuidiaceae Schimp.	*Thuidium kanedae* Sakurai	1
	Brachytheciaceae Schimp.	*Palamocladium euchloron* (Müll. Hal.) Wijk & Margad.	1
	Meteoriaceae Kindb.	*Meteorium polytrichum* Dozy & Molk.	1
		Trachypus humilis Lindb.	2
	Hypnaceae Schimp.	*Hypnum leptothallum* (Müll. Hal.) Paris	8
		Taxiphyllum taxirameum (Mitt.) M. Fleisch.	1
	Anomodontaceae Kindb.	*Anomodon minor* (Hedw.) Lindb.	1
Leizhuang Village, Guiyang City; E106°30'30"; N26°24'20"; 1150-1250m; 7 quadrats	*Grimmiaceae* Arn.	*Niphotrichum japonicum* (Dozy & Molk.) Bednarek-Ochyra & Ochyra	1

	Ptychomitriaceae Schimp.	*Ptychomitrium gardneri* Lesq.	1
	Pottiaceae Schimp.	*Barbula unguiculata* Hedw.	2
		Didymodon constrictus var. *constrictus* (Mitt.) Saito	1
		Didymodon fallax (Hedw.) R. H. Zander	6
		Hyophila involuta (Hook.) A. Jaeger	1
		Hyophila javanica (Nees & Blume) Brid.	1
		Hyophila nymaniana (M. Fleisch.) Menzel	1
		Trichostomum crispulum Bruch	1
	Bryaceae Schwägr.	*Bryum algovicum* Sendt.	1
		Bryum argenteum Hedw.	1
		Bryum calophyllum R. Br.	1
		Bryum funkii Schwägr.	2
		Bryum pallescens var. *pallescens* Schleich.	1
	Hypnaceae Schimp.	*Hypnum leptothallum* (Müll. Hal.) Paris	4
	Entodontaceae Kindb.	*Erythrodontrium julaceum* (Schwägr.) Paris	1

Supplemental Table S2 Five drought resistance indexes of two dominant mosses and climate data of their sampling sites

Sample	MP (%)	MAD (nmol/g)	Pro (μg/g)	SOD (U/g)	POD (U/min. g)	Average annual temperature （Max/min,°C）	Annual average sunshine (h)	Average annual rainfall (mm)	Elevation (m)	Longitude/Latitude
Hyp_Sl_1	18.60±6.17	6.284±5.776	15.422±5.776	284.27±7.806	1.368±0.133	16/14	1200-1300	1100-1200	1400	26°20'21"/106°47'34"
Hyp_Sl_2	19.19±2.22	5.890±0.925	14.709±4.848	280.09±16.723	1.193±0.108	16/14	1200-1300	1100-1200	1400	26°20'21"/106°47'34"
Hyp_Sl_3	17.08±2.92	6.742±7.043	15.813±3.214	269.31±10.260	1.005±0.098	16/14	1200-1300	1100-1200	1400	26°20'21"/106°47'34"
Hyp_Ve_1	12.28±5.20	5.031±3.596	17.085±5.147	307.82±8.062	1.587±0.302	14/12	1400	1500	1300	24°59'09"/104°54'27"
Hyp_Ve_2	15.79±1.21	5.064±3.017	16.354±2.415	300.37±10.782	1.569±0.074	14/12	1400	1500	1300	24°59'09"/104°54'27"
Hyp_Ve_3	12.58±4.62	4.541±6.178	17.189±1.785	303.11±8.479	1.635±0.487	14/12	1400	1500	1300	24°59'09"/104°54'27"
Hyp_Se_1	16.47±3.23	5.066±2.018	16.880±2.579	289.08±12.072	1.454±0.654	20/18	1100-1200	1100-1200	600	28°29'12"/108°32'47"
Hyp_Se_2	15.88±4.19	6.414±3.689	16.604±4.783	298.32±10.278	1.651±0.402	20/18	1100-1200	1100-1200	600	28°29'12"/108°32'47"
Hyp_Se_3	15.06±2.88	5.475±7.548	15.904±3.413	296.14±6.341	1.330±0.107	20/18	1100-1200	1100-1200	600	28°29'12"/108°32'47"
Hyo_Sl_1	19.17±2.26	6.291±1.774	9.914±5.785	211.24±11.257	0.965±0.065	16/14	1200-1300	1100-1200	1400	26°20'21"/106°47'34"
Hyo_Sl_2	20.52±2.67	6.752±5.241	10.090±6.751	222.34±8.014	0.885±0.982	16/14	1200-1300	1100-1200	1400	26°20'21"/106°47'34"
Hyo_Sl_3	19.66±5.33	6.631±1.581	9.885±5.422	203.25±15.354	1.007±0.360	16/14	1200-1300	1100-1200	1400	26°20'21"/106°47'34"
Hyo_Ve_1	16.02±3.29	5.943±3.687	12.654±3.552	252.34±17.102	1.211±0.324	14/12	1400	1500	1300	24°59'09"/104°54'27"
Hyo_Ve_2	19.28±6.14	5.821±3.147	10.817±2.987	247.363±9.014	1.364±0.645	14/12	1400	1500	1300	24°59'09"/104°54'27"
Hyo_Ve_3	17.57±2.31	6.372±1.079	10.895±4.058	248.234±7.820	1.225±0.087	14/12	1400	1500	1300	24°59'09"/104°54'27"
Hyo_Se_1	17.18±1.97	6.928±4.791	10.271±1.975	220.01±12.014	1.241±0.845	20/18	1100-1200	1100-1200	600	28°29'12"/108°32'47"
Hyo_Se_2	19.68±4.33	6.027±3.741	11.305±5.147	237.27±11.025	1.184±0.940	20/18	1100-1200	1100-1200	600	28°29'12"/108°32'47"
Hyo_Se_3	19.31±6.04	5.997±4.029	10.587±3.687	220.58±18.321	1.321±0.150	20/18	1100-1200	1100-1200	600	28°29'12"/108°32'47"

Supplemental Table S3

The bryophyte family compositions collected from all karst rocky desertification sites in Guizhou Province

No.	Family	Genera		Species	
		No.of genera	Percentage (%)	No.of species	Percentage (%)
1	*Pottiaceae*	11	19.64	32	22.07
2	*Bryaceae*	5	8.93	28	19.31
3	*Brachytheciaceae*	7	12.50	25	17.24
4	*Hypnaceae*	5	8.93	10	6.90
5	*Porellaceae*	1	1.79	6	4.14
6	*Mniaceae*	1	1.79	6	4.14
7	*Thuidiaceae*	2	3.57	6	4.14
8	*Anomodontaceae*	2	3.57	4	2.76
9	*Meteoriaceae*	3	5.36	4	2.76
10	*Entodontaceae*	2	3.57	4	2.76
11	*Aytoniaceae*	2	3.57	3	2.07
12	*Dicranaceae*	2	3.57	3	2.07
13	*Neckeraceae*	1	1.79	2	1.38
14	*Racopilaceae*	2	3.57	2	1.38
15	*Orthotrichaceae*	2	3.57	2	1.38
16	*Grimmiaceae*	2	3.57	2	1.38
17	*Lejeuneaceae*	1	1.79	1	0.69
18	*Ptychomitriaceae*	1	1.79	1	0.69
19	*Trachypodaceae*	1	1.79	1	0.69
20	*Bartramiaceae*	1	1.79	1	0.69
21	*Racopilaceae*	1	1.79	1	0.69
22	*Sematophyllaceae*	1	1.79	1	0.69
Total	--	56	100	145	100

Supplemental Table S4

Bacterial biomarkers sensitive to two dominant mosses at three karst rocky desertification types revealed by LEfSe analysis

Area	Phylum	Class	Order	Family	Genus	Enriched in species
**Ve**	*Actinobacteria*	*Actinobacteria*	*Corynebacteriales*	*Nocardiaceae*	*Smaragdicoccus*	
	Actinobacteria	*Actinobacteria*	*Frankiales*	*Geodermatophilaceae*	*Geodermatophilus*	
	Actinobacteria	*Actinobacteria*	*Micrococcales*	*Microbacteriaceae*	*Amnibacterium*	
	Bacteroidetes	*Cytophagia*	*Cytophagales*	*Cytophagaceae*	*Larkinella*	Hyp
	Chloroflexi	*Chloroflexia*	*Chloroflexales*	*Chloroflexaceae*	*Chloronema*	Hyp
	Cyanobacteria	*Cyanobacteria*	SubsectionIV			Hyo
	Cyanobacteria	*Cyanobacteria*	SubsectionIV	FamilyI		Hyo
	Cyanobacteria	*Cyanobacteria*	SubsectionIV	FamilyI	*Nostoc*	Hyo
	Proteobacteria	*Alphaproteobacteria*	*Rhizobiales*	*Methylobacteriaceae*	*Meganema*	
	Proteobacteria	*Alphaproteobacteria*	*Rhizobiales*	*Rhizobiales_Incertae_Sedis*	*Phreatobacter*	
	Proteobacteria	*Alphaproteobacteria*	*Rhodospirillales*	*Rhodospirillales_Incertae_Sedis*	*Geminicoccus*	
	Proteobacteria	*Betaproteobacteria*	*Burkholderiales*	*Comamonadaceae*	*Acidovorax*	Hyo
	Proteobacteria	*Deltaproteobacteria*	*Myxococcales*	VHS_B3_70		Hyp
	Proteobacteria	*Deltaproteobacteria*	*Myxococcales*	VHS_B3_70	norank	Hyp
	Proteobacteria	*Gammaproteobacteria*	*Pseudomonadales*			Hyo
	Proteobacteria	*Gammaproteobacteria*	*Pseudomonadales*	*Pseudomonadaceae*		Hyo
	Proteobacteria	*Gammaproteobacteria*	*Pseudomonadales*	*Pseudomonadaceae*	*Pseudomonas*	Hyo
**Se**	*Actinobacteria*	*Actinobacteria*	*Frankiales*			Hyp
	Actinobacteria	*Actinobacteria*	*Frankiales*	*Jatrophihabitans*		Hyp
	Actinobacteria	*Actinobacteria*	*Micromonosporales*	*Micromonosporaceae*	*Actinoplanes*	Hyo
	Chloroflexi	*Caldilineae*	*Caldilineales*	*Caldilineaceae*	unclassified	
	Cyanobacteria	*Cyanobacteria*	SubsectionIII	FamilyI	*Phormidium*	
	Proteobacteria	*Alphaproteobacteria*	*Alphaproteobacteria _Incertae_Sedis*	unclassified		Hyp
	Proteobacteria	*Alphaproteobacteria*	*Alphaproteobacteria _Incertae_Sedis*	unclassified	unclassified	Hyp
	Proteobacteria	*Alphaproteobacteria*	*Caulobacterales*	*Caulobacteraceae*	norank	
	Proteobacteria	*Alphaproteobacteria*	*Rhodospirillales*	*Rhodospirillaceae*	*Defluviicoccus*	
	Proteobacteria	*Deltaproteobacteria*	*Desulfovibrionales*			
	Proteobacteria	*Deltaproteobacteria*	*Desulfovibrionales*	*Desulfovibrionaceae*		Hyo
	Proteobacteria	*Deltaproteobacteria*	*Desulfovibrionales*	*Desulfovibrionaceae*	*Desulfovibrio*	Hyo
	Proteobacteria	*Deltaproteobacteria*	*Myxococcales*	mle1_27		Hyp
	Proteobacteria	*Deltaproteobacteria*	*Myxococcales*	mle1_28	norank	Hyp
	Proteobacteria	*Deltaproteobacteria*	*Myxococcales*	*Polyangiaceae*	*Sorangium*	Hyp
**Sl**	*Acidobacteria*	*Acidobacteria*	*Blastocatellales*	*Blastocatellaceae*	norank	Hyp
	Acidobacteria	*Acidobacteria*	*Propionibacteriales*	*Propionibacteriaceae*	unclassified	Hyp

continued Supplemental Table S4

Bacteroidetes	*Cytophagia*	*Cytophagales*	*Cytophagaceae*	*Huanghella*	Hyo
Bacteroidetes	*Cytophagia*	*Cytophagales*	*Cytophagaceae*	*Rudanella*	Hyo
Bacteroidetes	*Sphingobacteriia*	*Sphingobacteriales*	*Chitinophagaceae*	*Ferruginibacter*	Hyo
Bacteroidetes	*Sphingobacteriia*	*Sphingobacteriales*	*Chitinophagaceae*	*Segetibacter*	Hyo
Bacteroidetes	*Sphingobacteriia*	*Sphingobacteriales*	*Saprospiraceae*	*Phaeodactylibacter*	Hyo
Cyanobacteria	*Cyanobacteria*	SubsectionI			Hyp
Cyanobacteria	*Cyanobacteria*	SubsectionI	FamilyI		Hyp
Cyanobacteria	*Cyanobacteria*	SubsectionI	FamilyI	*Brasilonema*	Hyp
Cyanobacteria	*Cyanobacteria*	SubsectionI	FamilyI	*Gloeocalita*	Hyp
Cyanobacteria	*Cyanobacteria*	SubsectionIV	FamilyI	*Scytonema*	Hyp
Proteobacteria	*Alphaproteobacteria*	*Sphingomonadales*	*Sphingomonadaceae*	*Zymomonas*	Hyp
Proteobacteria	*Betaproteobacteria*	*Burkholderiales*			Hyo
Proteobacteria	*Betaproteobacteria*	*Burkholderiales*	*Comamonadaceae*		Hyo
Proteobacteria	*Betaproteobacteria*	*Burkholderiales*	*Comamonadaceae*	*Leptothrix*	Hyo
Proteobacteria	*Deltaproteobacteria*	*Myxococcales*	P3OB_42		Hyo
Proteobacteria	*Deltaproteobacteria*	*Myxococcales*	P3OB_42	norank	Hyo
Verrucomicrobia	*Spartobacteria*				Hyp
Verrucomicrobia	*Spartobacteria*	*Chthoniobacterales*			Hyp
Verrucomicrobia	*Spartobacteria*	*Chthoniobacterales*	unclassified		Hyp
Verrucomicrobia	*Spartobacteria*	*Chthoniobacterales*	unclassified	unclassified	Hyp

Supplemental Table S5

Alpha diversity index for bacteria and fungi associated with each sample

Type	Sample	Sequence number	OUT number	Chao estimator	Coverage	Shannon diversity	Simpson index
Bacteria	Hyp_Sl	60213	1441	1637.42±101.65	0.9871±0.010	5.7839±0.1666	0.0099±0.00254
	Hyp_Ve	54827	1626	1838.92±204.72	0.9815±0.014	6.0056±1.1112	0.0725±0.08247
	Hyp_Se	51787	1398	1714.78±379.41	0.9930±0.022	5.6792±0.4379	0.0256±0.01591
	Hyo_Sl	55696	1344	1966.55±125.29	0.9909±0.030	5.2378±0.4315	0.0492±0.00372
	Hyo_Ve	57708	1477	1731.79±65.10	0.9920±0.010	4.1148±0.2753	0.0170±0.00793
	Hyo_ Se	65144	1115	2139.68±261.19	0.9909±0.018	6.3372±0.5861	0.0185±0.01279
Fungi	Hyp_Sl	72704	855	1023.3±125.39	0.9972±0.045	3.9046±0.4830	0.0953±0.07812
	Hyp_Ve	61514	777	914.32±182.66	0.9974±0.028	3.7740±0.8910	0.10139±0.01222
	Hyp_Se	53166	502	639.95±344.26	0.9977±0.047	2.7524±1.1159	0.19644±0.02965
	Hyo_Sl	54839	764	923.07±244.18	0.9971±0.026	3.2787±0.8106	0.13591±0.08757
	Hyo_Ve	60746	609	778.78±102.78	0.9961±0.012	2.7022±0.7022	0.21859±0.12629
	Hyo_Se	64564	646	779.67±71.59	0.9974±0.024	2.5841±0.7143	0.23803±0.15308

Supplemental Table S6

Fungal biomarkers sensitive to two dominant mosses at three karst rocky desertification types revealed by LEfSe analysis

Area	Phylum	Class	Order	Family	Genus	Enriched in species
**Ve**	*Ascomycota*	*Dothideomycetes*	*Botryosphaeriales*	*Botryosphaeriaceae*	*Microdiplodia*	
	Ascomycota	*Dothideomycetes*	*Botryosphaeriales*	unclassified		
	Ascomycota	*Dothideomycetes*	*Botryosphaeriales*	unclassified	unclassified	
	Ascomycota	*Dothideomycetes*	*Capnodiales*	norank		
	Ascomycota	*Dothideomycetes*	*Capnodiales*	norank	*Rachicladosporium*	
	Ascomycota	*Dothideomycetes*			*Leptospora*	Hpy
	Ascomycota	*Dothideomycetes*	*Pleosporales*	*Cucurbitariaceae*	*Pyrenochaetopsis*	Hpy
	Ascomycota	*Dothideomycetes*	*Pleosporales*	norank	*Boeremia*	
	Ascomycota	*Dothideomycetes*	*Pleosporales*	norank	*Stagonosporopsis*	
	Ascomycota	*Dothideomycetes*	*Pleosporales*	*Phaeosphaeriaceae*	*Ophiosphaerella*	
	Ascomycota	*Dothideomycetes*	*Pleosporales*	*Phaeosphaeriaceae*	*Setophaeosphaeria*	
	Ascomycota	*Dothideomycetes*	*Pleosporales*	*Pleosporaceae*		Hyp
	Ascomycota	*Dothideomycetes*	*Pleosporales*	*Pleosporaceae*	*Dendryphion*	Hyp
	Ascomycota	*Dothideomycetes*	*Helotiales*	*Helotiaceae*		Hyp
	Ascomycota	*Dothideomycetes*	*Helotiales*	*Helotiaceae*	*Articulospora*	Hyp
	Ascomycota	norank			*Gyrothrix*	
	Ascomycota	*Sordariomycetes*	*Hypocreales*	norank		
	Ascomycota	*Sordariomycetes*	norank	norank		
	Ascomycota	*Sordariomycetes*	norank	norank	*Myrmecridium*	
	Ascomycota	*Sordariomycetes*	*Xylariales*	*Amphisphaeriaceae*	unclassified	
	Ascomycota	*Sordariomycetes*	*Xylariales*	norank		
	Ascomycota	*Sordariomycetes*	*Xylariales*	norank	*Monographella*	
	Basidiomycota	*Cystobasidiomycetes*				
	Basidiomycota	*Cystobasidiomycetes*	*Erythrobasidiales*			Hyp
	Basidiomycota	*Cystobasidiomycetes*	*Erythrobasidiales*	norank		Hyp
	Basidiomycota	*Cystobasidiomycetes*	*Erythrobasidiales*	norank	*Erythrobasidium*	Hyp
	Basidiomycota	*Microbotryomycetes*				
	Basidiomycota	*Microbotryomycetes*	*Sporidiobolales*	norank		
	Basidiomycota	*Microbotryomycetes*	*Sporidiobolales*	norank	norank	
	Chytridiomycota	*Chytridiomycetes*	*Rhizophlyctidales*			
	Chytridiomycota	*Chytridiomycetes*	*Rhizophlyctidales*	unclassified		
	Chytridiomycota	*Chytridiomycetes*	*Rhizophlyctidales*	unclassified	unclassified	
**Sl**	*Ascomycota*	*Dothideomycetes*	*Capnodiales*	*Mycosphaerellaceae*	*Mycosphaerella*	
	Ascomycota	*Dothideomycetes*	*Capnodiales*	*Teratosphaeriaceae*		
	Ascomycota	*Dothideomycetes*	*Capnodiales*	*Teratosphaeriaceae*	*Catenulostroma*	Hyo
	Ascomycota	*Dothideomycetes*	*Capnodiales*	*Teratosphaeriaceae*	*Devriesia*	

continued Supplemental Table S6

Ascomycota	*Dothideomycetes*	*Capnodiales*	unclassified		
Ascomycota	*Dothideomycetes*	*Capnodiales*	unclassified	unclassified	
Ascomycota	*Dothideomycetes*	*Pleosporales*	*Montagnulaceae*		
Ascomycota	*Dothideomycetes*	*Pleosporales*	unclassified		
Ascomycota	*Dothideomycetes*	*Pleosporales*		*Montagnulaceae*	
Ascomycota	*Dothideomycetes*	*Pleosporales*		*Pyrenochaeta*	Hyo
Ascomycota	*Dothideomycetes*	*Pleosporales*	*Pleosporaceae*	*Curvularia*	
Ascomycota	*Eurotiomycetes*	unclassified	unclassified		
Ascomycota	*Eurotiomycetes*	unclassified	unclassified	unclassified	
Ascomycota	*Eurotiomycetes*	unclassified			
Ascomycota	*Eurotiomycetes*	*Verrucariales*	*Verrucariaceae*	*Verrucaria*	
Ascomycota	*Lecanoromycetes*				
Ascomycota	*Lecanoromycetes*	*Lecanorales*	*Ramalinaceae*		
Ascomycota	*Lecanoromycetes*	*Lecanorales*	unclassified		
Ascomycota	*Lecanoromycetes*	*Lecanorales*	unclassified	unclassified	
Ascomycota	*Lecanoromycetes*	*Lecanorales*			
Ascomycota	*Lecanoromycetes*	*Ostropales*			
Ascomycota	*Leotiomycetes*	*Helotiales*	*Dermateaceae*		
Ascomycota	*Leotiomycetes*	*Helotiales*	norank	*Trimmatostroma*	
Ascomycota	norank			*Pseudorobillarda*	
Ascomycota	*Orbiliomycetes*	*Orbiliales*			Hyo
Ascomycota	*Orbiliomycetes*	*Orbiliales*	*Orbiliaceae*		Hyo
Ascomycota	*Orbiliomycetes*	*Orbiliales*	*Orbiliaceae*	*Orbiliaceae*	Hyo
Ascomycota	*Sordariomycetes*	*Hypocreales*		*Stilbella*	
Ascomycota	*Sordariomycetes*	*Sordariales*	*Cephalothecaceae*		
Ascomycota	*Sordariomycetes*	*Sordariales*	*Cephalothecaceae*	unclassified	
Ascomycota	*Sordariomycetes*	*Sordariales*	*Cephalothecaceae*	*Humicola*	
Ascomycota	*Sordariomycetes*	*Sordariales*	*Lasiosphaeriaceae*	*Podospora*	
Ascomycota	*Sordariomycetes*	*Xylariales*	*Amphisphaeriaceae*	*Monochaetia*	Hyp
Ascomycota	*Taphrinomycetes*				Hyo
Ascomycota	*Taphrinomycetes*	*Taphrinales*			Hyo
Ascomycota	*Taphrinomycetes*	*Taphrinales*	*Taphrinales*		Hyo
Ascomycota	*Taphrinomycetes*	*Taphrinales*	*Taphrinales*	*Taphrina*	Hyo
Basidiomycota	*Agaricomycetes*				Hyo
Basidiomycota	*Agaricomycetes*	*Agaricales*	*Agaricaceae*	*Lycoperdon*	Hyo
Basidiomycota	*Agaricomycetes*	*Agaricales*	*Strophariaceae*	*Hypholoma*	Hyo
Basidiomycota	*Agaricomycetes*	*Atheliales*	*Atheliaceae*	*Amphinema*	Hyo
Basidiomycota	*Agaricomycetes*	*Boletales*			
Basidiomycota	*Agaricomycetes*	*Boletales*	*Paxillaceae*		
Basidiomycota	*Agaricomycetes*	*Boletales*	*Paxillaceae*	*Gyrodon*	

	Basidiomycota	*Agaricomycetes*	*Russulales*	*Stereaceae*		
	Basidiomycota	*Agaricomycetes*	*Russulales*	*Stereaceae*	*Gloeocystidiellum*	Hyo
	Basidiomycota	*Agaricomycetes*	*Thelephorales*	*Thelephoraceae*	*Tomentella*	
	Basidiomycota	*Microbotryomycetes*	*Sporidiobolales*		*Rhodotorula*	Hyp
	Basidiomycota	*Pucciniomycetes*	*Septobasidiales*			
	Basidiomycota	*Pucciniomycetes*	*Septobasidiales*	*Septobasidiaceae*		
	Basidiomycota	*Pucciniomycetes*	*Septobasidiales*	*Septobasidiaceae*	unclassified	
**Se**	*Ascomycota*	*Lecanoromycetes*	*Lecanorales*	*Ramalinaceae*	*Bacidia*	
	Ascomycota	*Sordariomycetes*	*Hypocreales*	*Ophiocordycipitaceae*	unclassified	Hyp
	Basidiomycota	*Agaricomycetes*	*Polyporales*	*Fomitopsidaceae*	*Fibroporia*	
	Basidiomycota	*Agaricomycetes*	*Polyporales*	*Polyporaceae*	*Trametes*	Hyp
	Basidiomycota	*Tremellomycetes*	*Tremellales*		*Hannaella*	

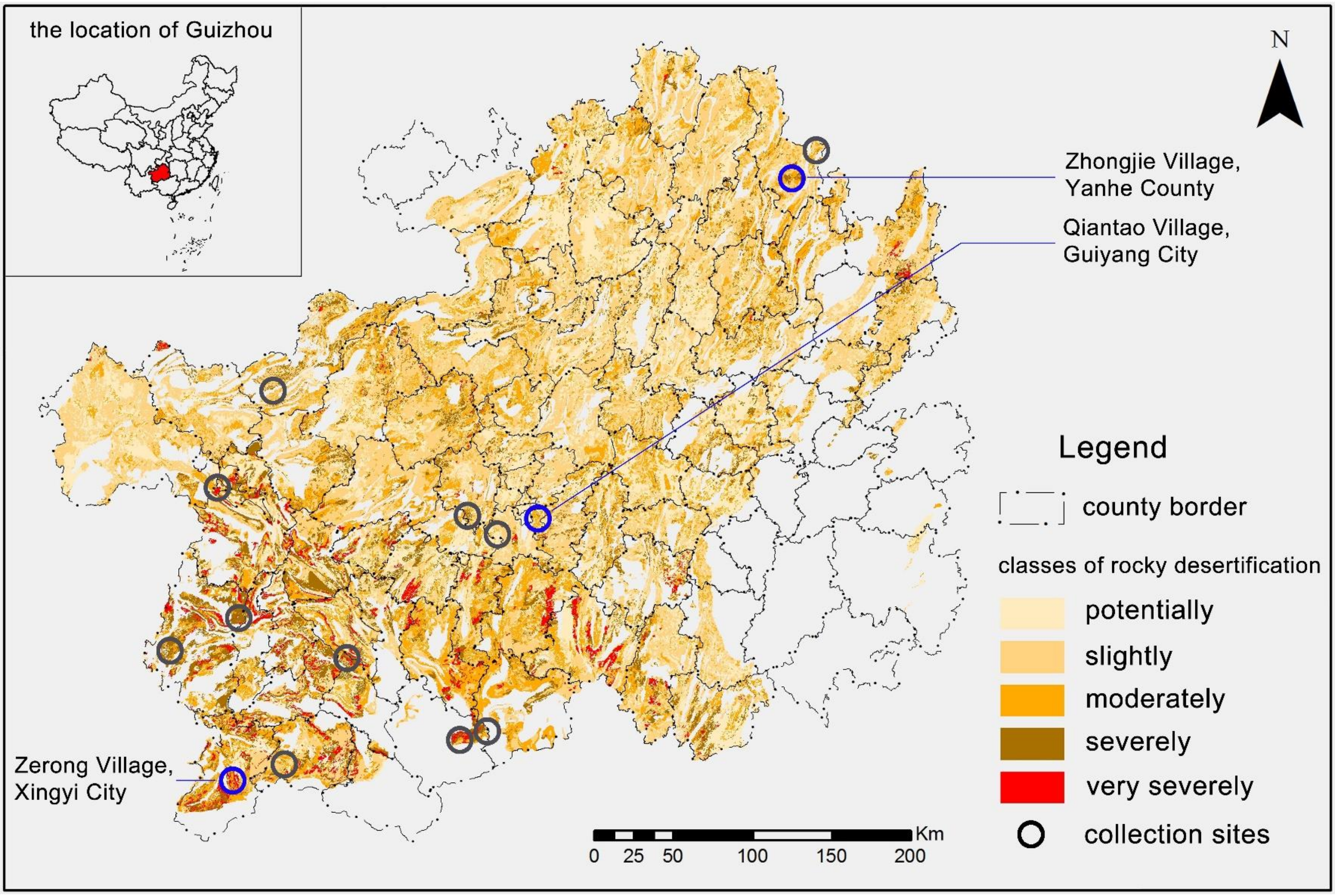

Supplemental Fig. S1 The main classes and horizontal distribution of rocky desertification areas in Guizhou province and the geographic location of all sample sites. All circles represent the location of moss-collecting sites, and the blue circle represents the collection sites of moss-related microbial samples. Three sites located in Xingyi, Yanhe county and Guiyang city, corresponding to the very severely, the severely and the slightly areas, respectively. Two moss species were set up in triplicate plots in each collection sites.

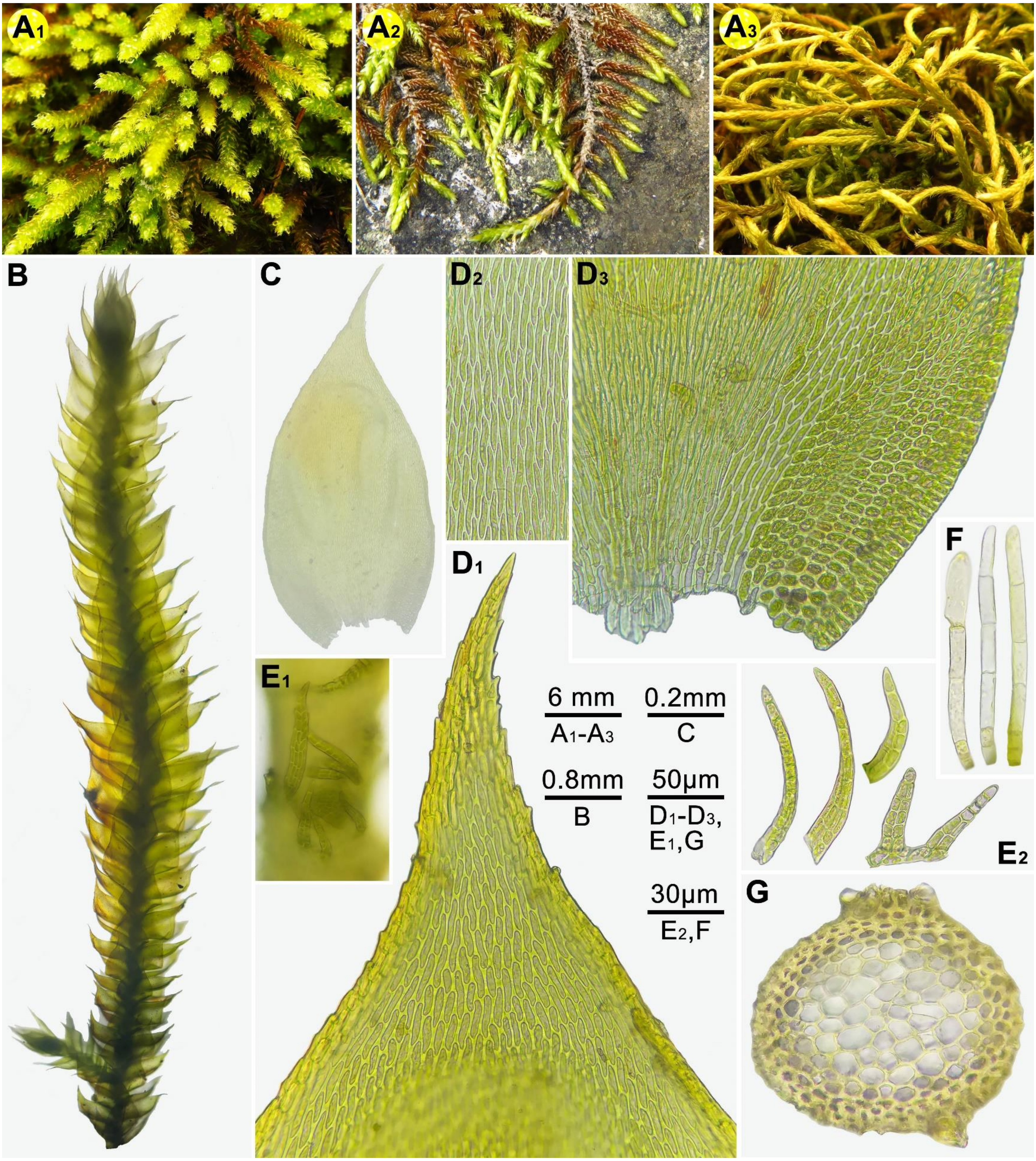

Supplemental Fig. S2 *Hypnum leptothallum* (Müll. Hal.) Paris, A_1-A_3. Appearance with different water content in field, A_1 showing fully obsorbed water, A_2 showing earlier stage of drought stress, A_3 showing several days after drought stress; B. Portion of shoot showing densely imbricated leaves; C. Obviously concaved leaf to reserve more water; D_1. Apical cells of leaf; D_2. Median cells of leaf; D_3. Basal cells of leaf; E_1-E_2. Pseudoparaphyllium, E_1 showing clustered Pseudoparaphyllia on the stem to protect the young buds; F. Axillary hairs showing inflated cells to store water; F. Transverse section of stem showing central aqueduct thin-walled cells and external thick-walled cells.

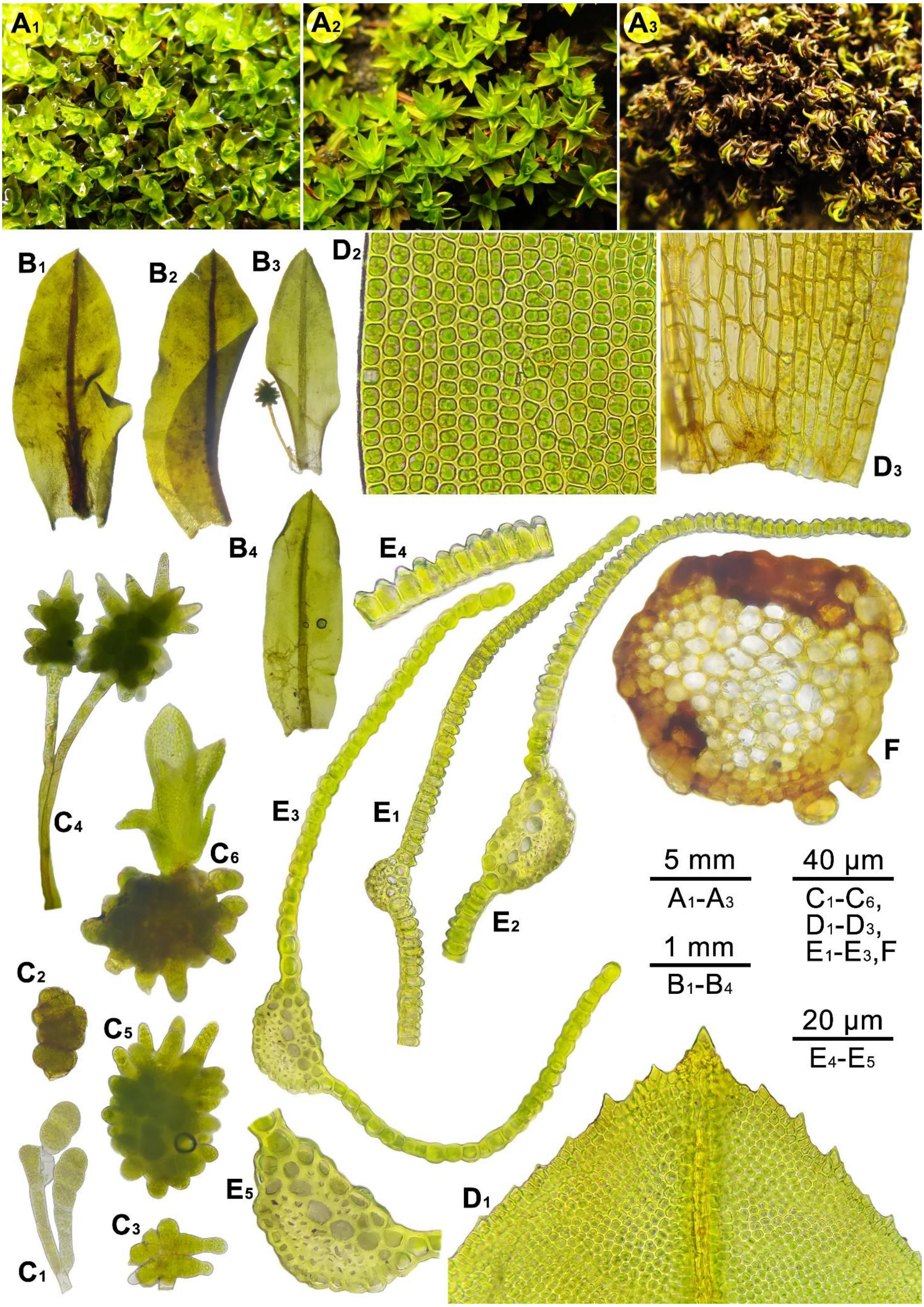

Supplemental Fig. S3 *Hyophila involuta* (Hook.) A. Jaeger, A_1-A_3. Appearance with different water content in field, A_1 showing fully obsorbed water, A_2 showing earlier stage of drought stress, A_3 showing several days after drought stress; B_1-B_2, Leaves; C_1-C_6. Gemmae at different developmental stages, C_6 showing a young plant; D_1. Apical cells of leaf; D_2. Median cells of leaf; D_3. Basal cells of leaf; E_1-E_3. Transeverse section of leaves; E_4. Portion of leaf transeverse section showing mamilla on cell surface; E_5. Transeverse section of costa showing enlarged central aqueduct cells; F. Transverse section of stem.

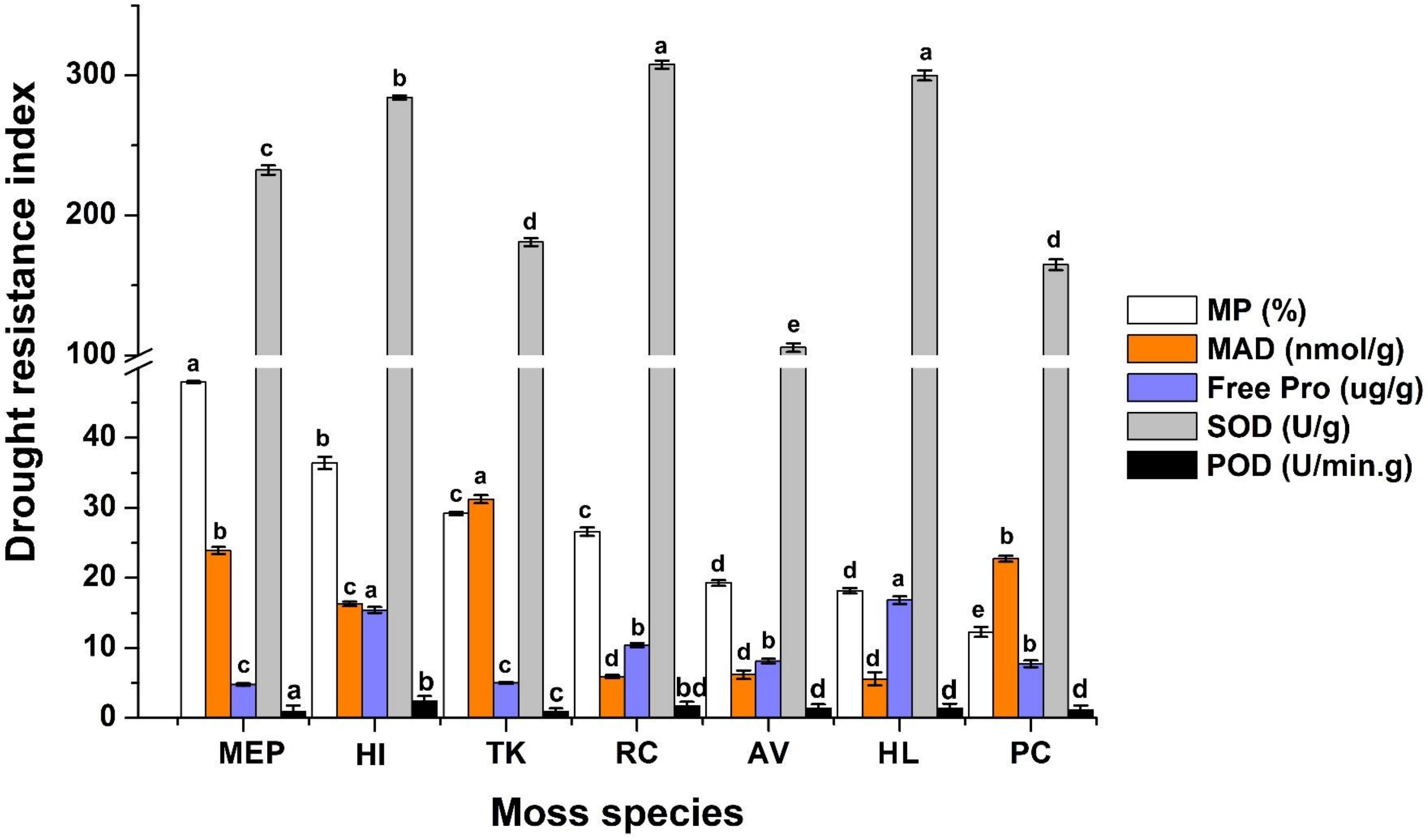

Supplemental Fig. S4 Five drought resistance indexes of 7 moss species. MP, membrane permeability; MAD, malondialdehyde; Pro, free proline; SOD, SOD activity; POD, POD activity. MEP, *M. polytrichum*; HI, *H. involuta*; TK, *T. kanedae*; RC, *R. cuspidigerum*; AV, *A. viticulosus*; HL, *H. leptothallum*; PC, *P. cuspidatum*. Different letters marked between the two groups in the same measurement index indicated the significant difference (P<0.05).

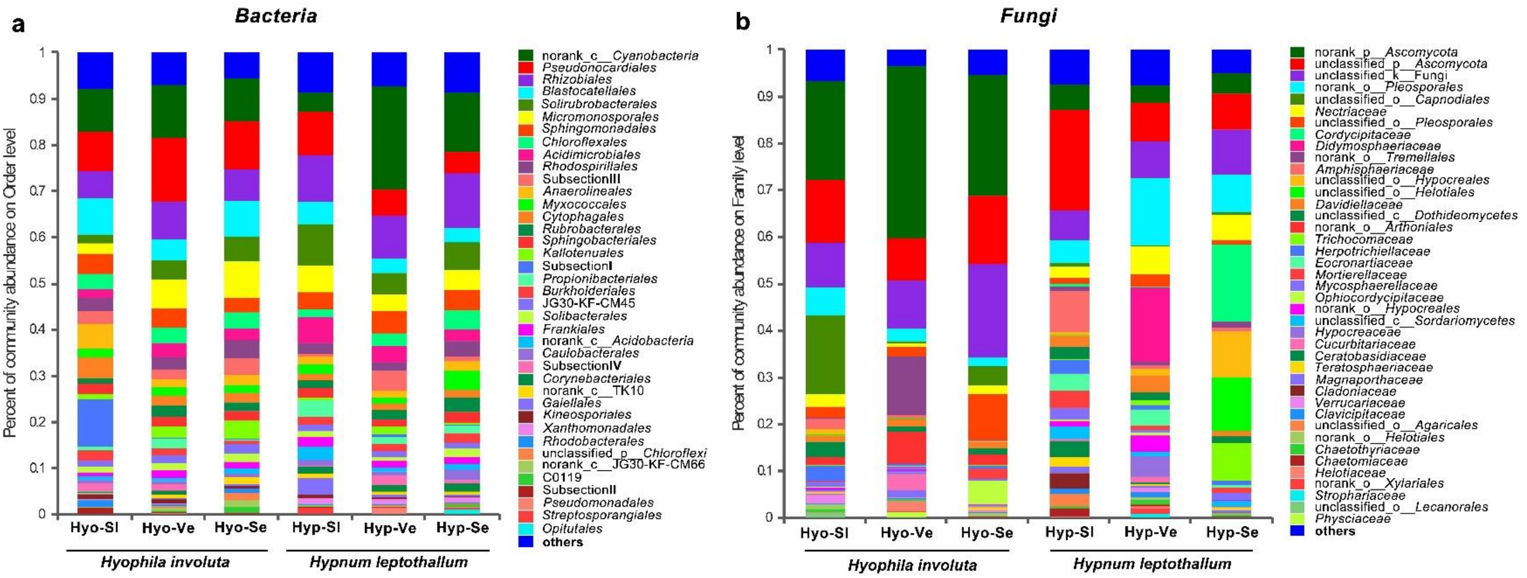

Supplemental Fig. S5 Relative abundance of the taxonomic composition of the bacterial (**a**) and fungal (**b**) communities of two mosses at order and family levels. The two mosses including *H. involute* (Hyo) and *H. leptothallum* (Hyp), were collected from very severely areas, severely areas and slightly areas. In (A) and (B), class names were colour-coded on the right with respective taxonomic names listed above.

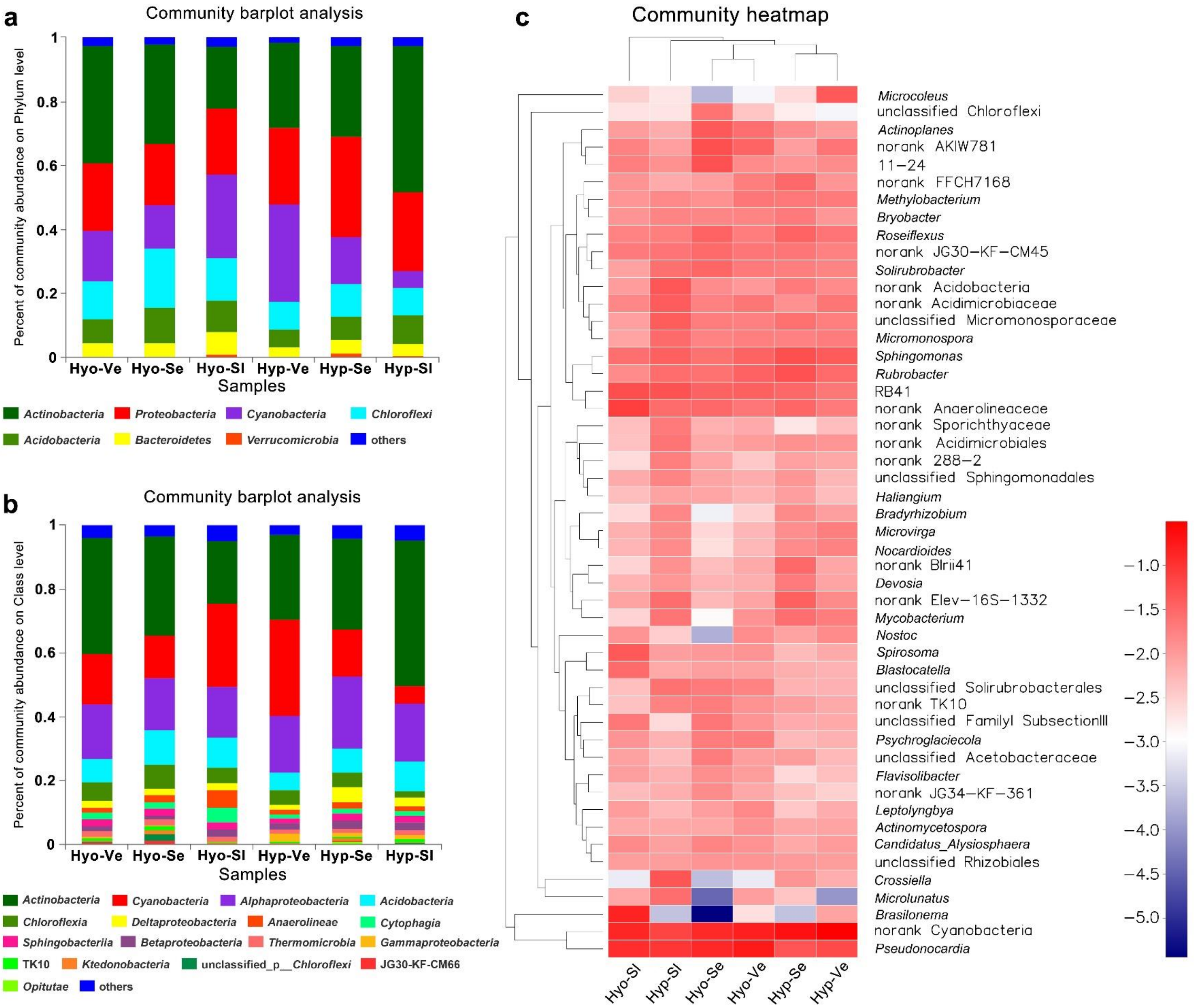

Supplemental Fig. S6 (a) and (b) Relative abundance of the taxonomic composition of the bacterial communities of two mosses at phylum and class levels, respectively and, (c) the heatmap diagram of the dominant 50 genera under three karst rocky desertification areas. The two mosses including *H. involute* (Hyo) and *H. leptothallum* (Hyp), were collected from very severely areas, severely areas and slightly areas.

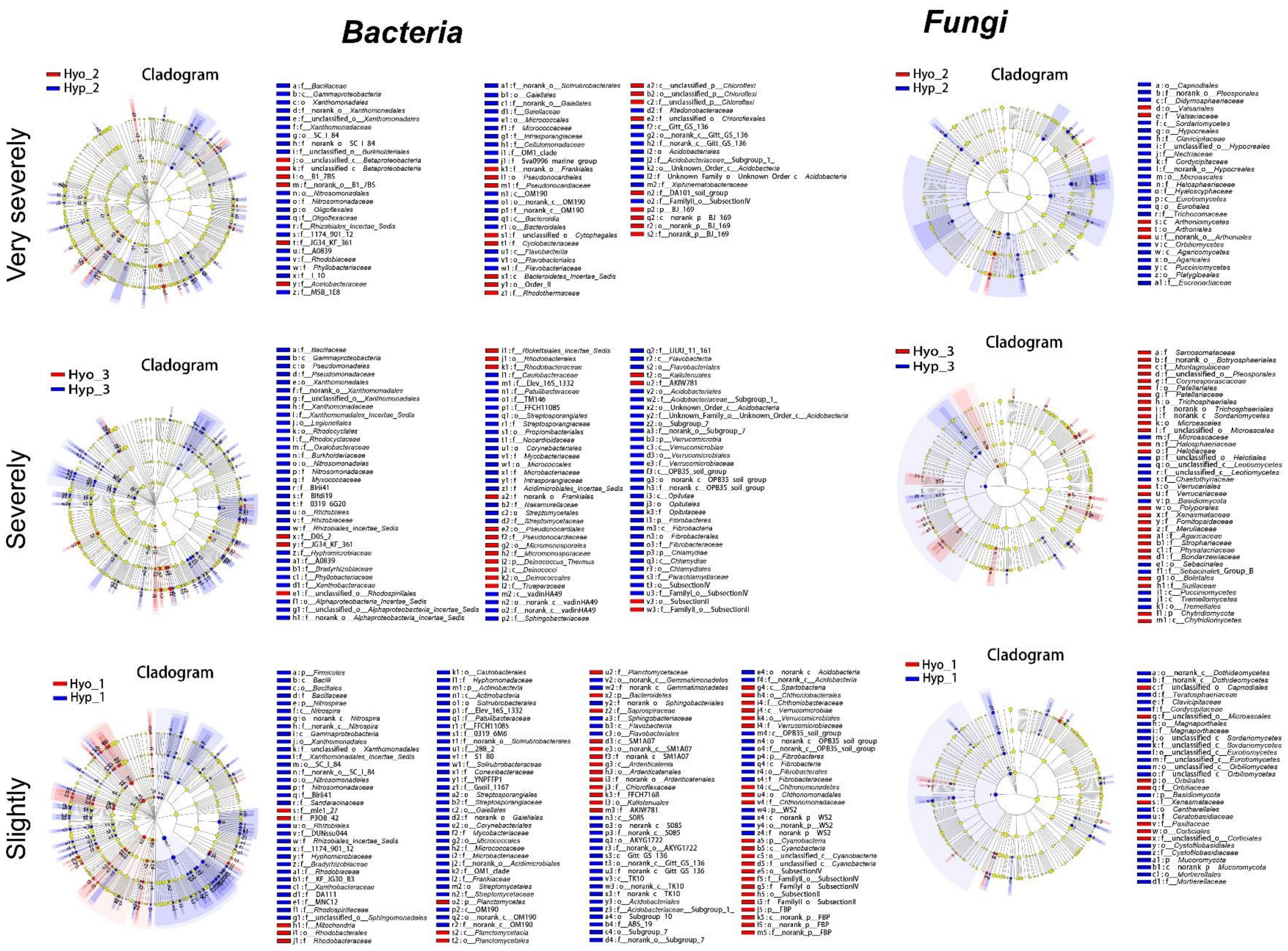

Supplemental Fig. S7 LEfSe results revealed bacteria (from phylum level to order level) and fungi biomarkers (from phylum level to family level) that were sensitive to three karst rocky desertification areas (slightly, severely and very severely) of *H. involute* (Hyo) and *H. leptothallum* (Hyp), respectively.

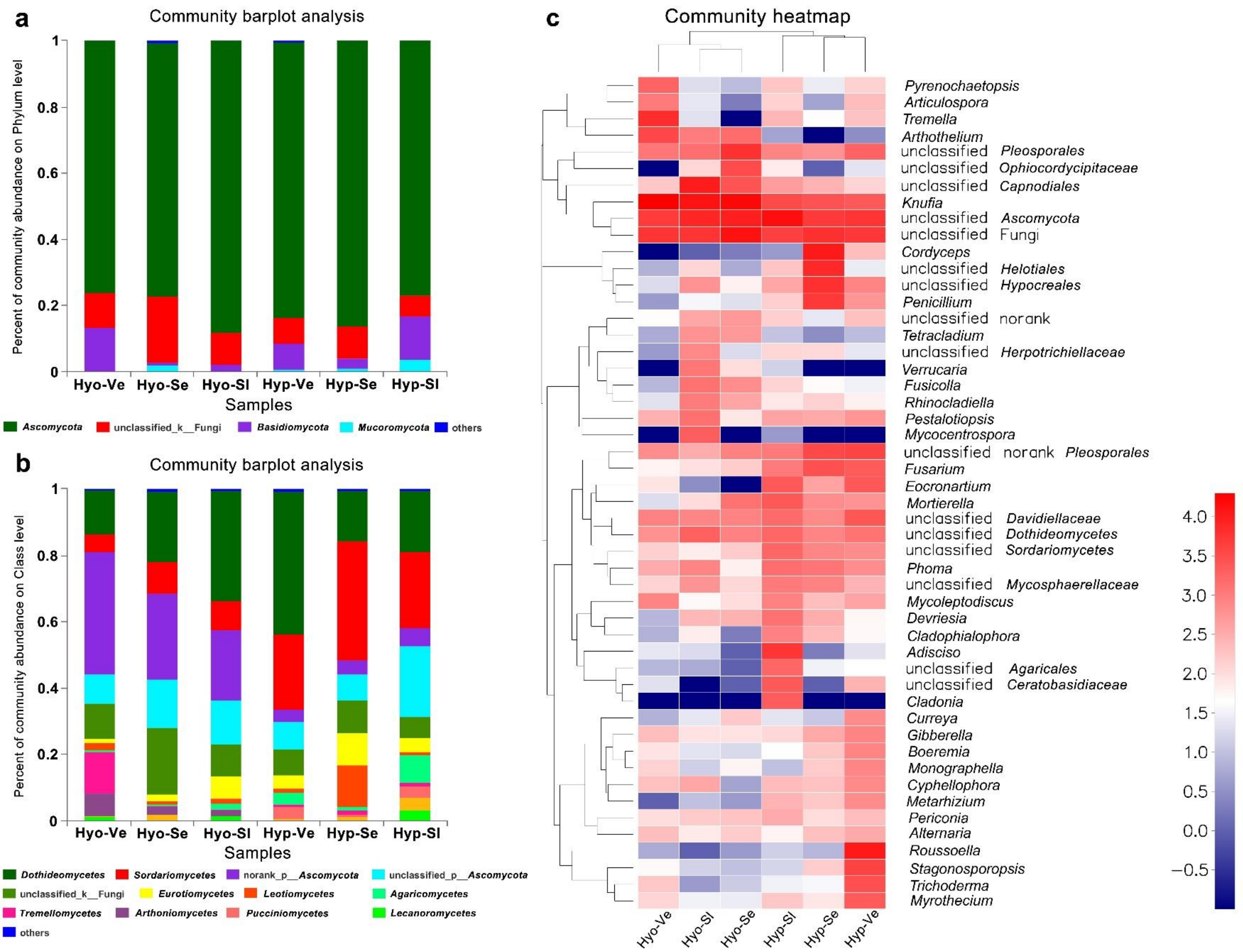

Supplemental Fig. S8 (a) and (b) Relative abundance of the taxonomic composition of the fungal communities of two mosses at phylum and class levels, respectively and, (c) the heatmap diagram of the dominant 50 genera under three karst rocky desertification areas. The two mosses including *H. involute* (Hyo) and *H. leptothallum* (Hyp), were collected from very severely areas, severely areas and slightly areas.

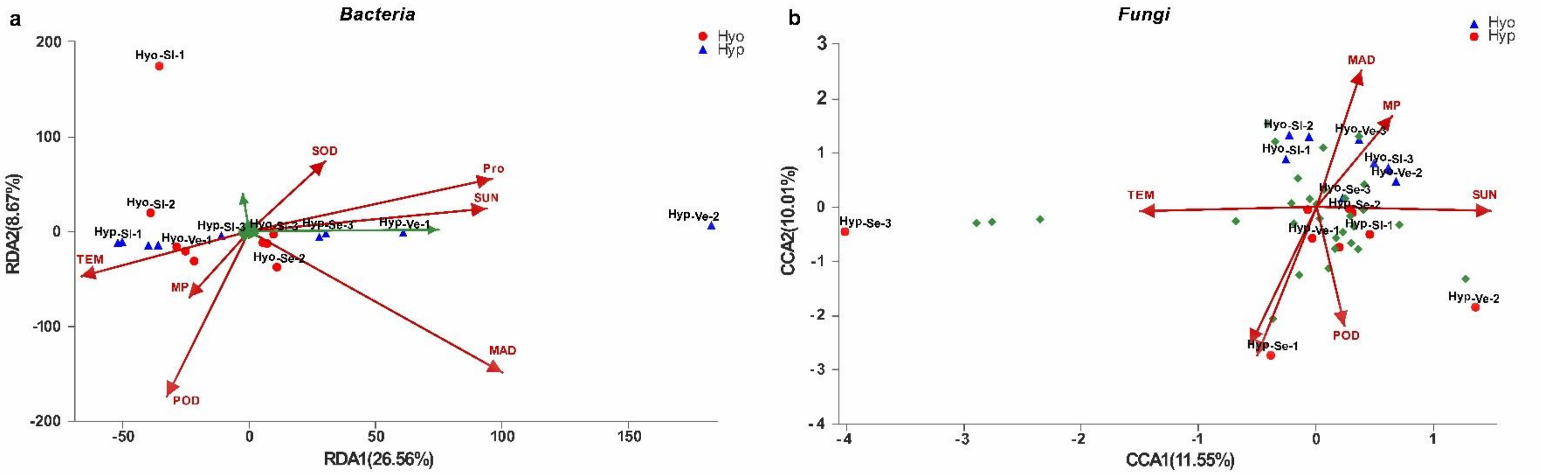

Supplemental Fig. S9 Redundancy analysis to show the correlation between bacterial and fungal communities and drought resistance properties of two moss species under the three karst rocky desertification types. The two mosses including *H. involute* (Hyo) and *H. leptothallum* (Hyp), were collected from very severely areas, severely areas and slightly areas. (**a**) RDA analysis of bacterial community; (**b**) CCA analysis of fungal community.
